# Sex-associated differences in frequencies and prognostic impact of recurrent genetic alterations in adult acute myeloid leukemia (Alliance, AMLCG)

**DOI:** 10.1038/s41375-023-02068-8

**Published:** 2023-11-28

**Authors:** Michael Ozga, Deedra Nicolet, Krzysztof Mrózek, Ayse S. Yilmaz, Jessica Kohlschmidt, Karilyn T. Larkin, James S. Blachly, Christopher C. Oakes, Jill Buss, Christopher J. Walker, Shelley Orwick, Vindi Jurinovic, Maja Rothenberg-Thurley, Annika Dufour, Stephanie Schneider, Maria Cristina Sauerland, Dennis Görlich, Utz Krug, Wolfgang E. Berdel, Bernhard J. Woermann, Wolfgang Hiddemann, Jan Braess, Marion Subklewe, Karsten Spiekermann, Andrew J. Carroll, William G. Blum, Bayard L. Powell, Jonathan E. Kolitz, Joseph O. Moore, Robert J. Mayer, Richard A. Larson, Geoffrey L. Uy, Wendy Stock, Klaus H. Metzeler, H. Leighton Grimes, John C. Byrd, Nathan Salomonis, Tobias Herold, Alice S. Mims, Ann-Kathrin Eisfeld

**Affiliations:** 1 The Ohio State University Comprehensive Cancer Center, Columbus, OH USA; 2 The Ohio State University Comprehensive Cancer Center, Clara D. Bloomfield Center for Leukemia Outcomes Research, Columbus, OH USA; 3 Alliance Statistics and Data Management Center, The Ohio State University Comprehensive Cancer Center, Columbus, OH USA; 4 Laboratory for Leukemia Diagnostics, Department of Medicine III, University Hospital, LMU Munich, Munich, Germany; 5 Institute of Human Genetics, University Hospital, LMU Munich, Munich, Germany; 6 Institute of Biostatistics and Clinical Research, University of Münster, Münster, Germany; 7 Department of Medicine 3, Klinikum Leverkusen, Leverkusen, Germany; 8 Department of Medicine, Hematology and Oncology, University of Münster, Münster, Germany; 9 German Society of Hematology and Oncology, Berlin, Germany; 10 German Cancer Research Center (DKFZ), Heidelberg, Germany; 11 Department of Oncology and Hematology, Hospital Barmherzige Brüder, Regensburg, Germany; 12 German Cancer Consortium (DKTK), Partner Site Munich, Munich, Germany; 13 Department of Genetics, University of Alabama at Birmingham, Birmingham, AL USA; 14 Emory University School of Medicine, Atlanta, GA USA; 15 Wake Forest University Health Sciences, Winston-Salem, NC USA; 16 Monter Cancer Center, Hofstra Northwell School of Medicine, Lake Success, NY USA; 17 Duke Cancer Institute, Duke University Health System, Durham, NC USA; 18 Department of Medical Oncology, Dana-Farber/Partners CancerCare, Boston, MA USA; 19 University of Chicago Medical Center, Chicago, IL USA; 20 Division of Oncology, Washington University School of Medicine, St. Louis, MO USA; 21 Department of Hematology, Cellular Therapy, and Hemostaseology, Leipzig University Hospital, Leipzig, Germany; 22 Division of Immunobiology, Cincinnati Children’s Hospital, University of Cincinnati, Cincinnati, OH USA; 23 Department of Pediatrics, University of Cincinnati, Cincinnati, OH USA; 24 Department of Internal Medicine, University of Cincinnati, Cincinnati, OH USA; 25 Division of Biomedical Informatics, Cincinnati Children’s Hospital, University of Cincinnati, Cincinnati, OH USA

**Keywords:** Acute myeloid leukaemia, Genetics research

## Abstract

Clinical outcome of patients with acute myeloid leukemia (AML) is associated with demographic and genetic features. Although the associations of acquired genetic alterations with patients’ sex have been recently analyzed, their impact on outcome of female and male patients has not yet been comprehensively assessed. We performed mutational profiling, cytogenetic and outcome analyses in 1726 adults with AML (749 female and 977 male) treated on frontline Alliance for Clinical Trials in Oncology protocols. A validation cohort comprised 465 women and 489 men treated on frontline protocols of the German AML Cooperative Group. Compared with men, women more often had normal karyotype, *FLT3*-ITD, *DNMT3A*, *NPM1* and *WT1* mutations and less often complex karyotype, *ASXL1*, *SRSF2*, *U2AF1*, *RUNX1*, or *KIT* mutations. More women were in the 2022 European LeukemiaNet intermediate-risk group and more men in adverse-risk group. We found sex differences in co-occurring mutation patterns and prognostic impact of select genetic alterations. The mutation-associated splicing events and gene-expression profiles also differed between sexes. In patients aged <60 years, *SF3B1* mutations were male-specific adverse outcome prognosticators. We conclude that sex differences in AML-associated genetic alterations and mutation-specific differential splicing events highlight the importance of patients’ sex in analyses of AML biology and prognostication.

## Introduction

Acute myeloid leukemia (AML) is a biologically and clinically heterogenous disease that affects a diverse patient population composed of both sexes and all ages [1, 2]. Several pretreatment factors, both disease- and patient-specific, affect prognosis of AML patients [1–19]. The former include recurrent cytogenetic findings [3–11] and gene mutations [12–19] at diagnosis, whereas increasing age is a well-known patient-specific predictor of worse survival [1, 2, 9, 20]. The incidence of acquired cytogenetic and molecular alterations and their prognostic impact vary by age [9] and racial-ethnic identity [21, 22]. Moreover, AML is more common in men than women [1, 23], but the reasons for this sex bias remain largely unknown. Despite the recognition of differences in frequencies of genetic alterations in male and female patients with AML [23, 24], to our knowledge, no large study has yet comprehensively assessed potential associations between patient sex and prognostic impact of recurrent mutations. Therefore, we assessed the frequencies of pretreatment cytogenetic and molecular features and their impact on outcomes of male and female patients in a cohort of 1726 adults with AML in the USA and in a large validation cohort of 954 patients in Germany, both treated on multicenter studies.

## Methods

### Patients and treatment

We analyzed 1726 adults with de novo AML, including 749 self-reported women and 977 men, who were treated on frontline Cancer and Leukemia Group B (CALGB)/Alliance for Clinical Trials in Oncology (Alliance) protocols. We limited our study to patients with de novo AML in order to avoid the confounding effects of AML type, that is, de novo AML versus AML evolving from an antecedent myelodysplastic syndrome versus therapy-related AML. Self-reported patient sex was confirmed by the results of centrally reviewed [25] metaphase karyotyping. Almost all patients received intensive cytarabine/anthracycline-based frontline treatment on CALGB/Alliance trials between 1986 and 2015 (details are provided in the Supplementary Information). Per study protocols, no patient received an allogeneic hematopoietic stem-cell transplantation (HSCT) in first complete remission (CR), and patients who received an off-protocol HSCT were excluded from analyses of disease-free (DFS) and overall survival (OS) because their follow-up data were either missing or incomplete. Because of differences in the treatment protocols between patients aged <60 years and those aged 60 years and older (Supplementary Information), we performed outcome analyses separately for these age groups. Institutional Review Board approval of all CALGB/Alliance and German AML Cooperative Group (AMLCG) protocols was obtained before any research was performed. Patients provided study-specific written informed consent to participate in treatment studies. Treatment protocols were in accordance with the Declaration of Helsinki. The AMLCG validation cohort of 954 patients (465 female and 489 male) is described in the Supplementary Information.

### Mutational profiling

The mutational status of 80 protein-coding genes was determined centrally at The Ohio State University by targeted amplicon sequencing using the MiSeq platform (Illumina, San Diego, CA) [26]. Testing for *FLT3* internal tandem duplications (*FLT3*-ITD) was performed with the Sanger sequencing method [27]. Determination of *CEBPA*^bZIP^ status was done following 2022 European LeukemiaNet (ELN) guidelines [1] using Miseq panel and/or transcriptional profiling [28]. *SF3B1* and *SRSF2* were considered as mutated if they concurred with the biology-associated differential splicing events (see details below). Experimental details are provided in the Supplementary Information. Preparation of samples and sequencing in the AMLCG validation cohort followed a comparable workflow [18]. Methods used to analyze gene expression and differential splicing are described in the  Supplementary Information.

### Clinical endpoints and statistical analysis

Definitions of clinical endpoints—CR, early death, DFS and OS—are provided in the Supplementary Information [29]. Pretreatment features of female and male patients were compared using the Fisher’s exact for categorical variables and Wilcoxon rank-sum tests for continuous variables. Estimated probabilities of DFS and OS were calculated using the Kaplan–Meier method, and the log-rank test evaluated differences between survival distributions [30]. We used logistic regression for modeling CR and Cox proportional hazard regression for interaction and modeling DFS and OS for univariable and multivariable outcome analyses, which were calculated using a limited backward selection technique, and adjusted *P* values to control for per family error rate. All analyses were performed by the Alliance Statistics and Data Center on a database locked on June 6, 2021, using SAS 9.4, TIBCO Spotfire S+ 8.2 and GraphPad Prism 9.

## Results

### Clinical and molecular characteristics of AML patients with respect to sex

Our analysis of pretreatment characteristics in the CALGB/Alliance cohort revealed that female patients tended to be younger (median age, 51 vs 54 years, *P* = 0.06), and had higher white blood cell counts (median, 26.0 vs 22.2 × 10^9^/L, *P* = 0.009) and percentages of bone marrow blasts (median, 68% vs 65%, *P* = 0.04). Females had also more often cytogenetically normal AML (CN-AML; 52% vs 42%, *P* < 0.001), and less often complex karyotype (8% vs 12%, *P* = 0.005; Supplementary Table 1). Mutational analysis revealed that female patients harbored more often *DNMT3A* (*P* < 0.001), *NPM1* (*P* < 0.001) and *WT1* (*P* = 0.02) mutations as well as *FLT3*-ITD (*P* = 0.03), and less often *ASXL1* (*P* < 0.001), *SRSF2* (*P* < 0.001), *U2AF1* (*P* = 0.001), *RUNX1* (*P* = 0.04), or *KIT* (*P* = 0.05) mutations (Table 1). Notably, all aforementioned genes are located in autosomal chromosomes. We also observed sex-associated differences in the frequencies of mutations in genes categorized into the major AML-associated functional groups, that included female patients having a higher frequency of mutations in methylation-related genes (51% vs 46%, *P* = 0.02) and male patients having a higher frequency of mutations in genes involving chromatin remodeling (23% vs 18%, *P* = 0.02), spliceosome (22% vs 11%, *P* < 0.001), and, by trend, transcription factors (28% vs 24%, *P* = 0.06); men also harbored myelodysplasia-related gene mutations more often than women (37% vs 27%, *P* < 0.001; Table 2).

**Table 1:** Frequencies of mutations in single genes in female and male patients with AML treated on the CALGB/Alliance frontline protocols and those treated on the German AML Cooperative Group frontline protocols.

Gene	CALGB/Alliance patients			AMLCG patients		
	Females *n* = 749	Males *n* = 977	P*	Females *n* = 465	Males *n* = 489	P*
*ASXL1*, *n* (%)			<0.001			<0.001
Mutated	40 (5)	114 (12)		19 (4)	79 (16)	
Wild-type	709 (95)	863 (88)		446 (96)	410 (84)	
*BCOR*, *n* (%)			0.92			0.08
Mutated	49 (7)	62 (6)		26 (6)	42 (9)	
Wild-type	700 (93)	915 (94)		439 (94)	447 (91)	
*BCORL1*, *n* (%)			0.15			0.56
Mutated	28 (4)	24 (2)		11 (2)	15 (3)	
Wild-type	721 (96)	953 (98)		454 (98)	474 (97)	
*CBL*, *n* (%)			0.87			0.09
Mutated	15 (2)	21 (2)		7 (2)	16 (3)	
Wild-type	734 (98)	956 (98)		458 (98)	473 (97)	
*CEBPA*^bZIP^, *n* (%)			0.29			
Present	46 (6)	63 (7)				
Absent	672 (94)	853 (93)				
*DNMT3A*, *n* (%)			<0.001			<0.001
Mutated	221 (30)	186 (19)		200 (43)	148 (30)	
Wild-type	528 (70)	791 (81)		265 (57)	341 (70)	
*EZH2*, *n* (%)			0.18			0.005
Mutated	16 (2)	32 (3)		10 (2)	28 (6)	
Wild-type	733 (98)	945 (97)		455 (98)	461 (94)	
*FBXW7*, *n* (%)			0.19			1.00
Mutated	2 (<1)	0 (0)		1 (<1)	1 (<1)	
Wild-type	744 (99)	975 (100)		464 (99)	488 (99)	
*FLT3*-ITD, *n* (%)			0.03			0.03
Present	192 (27)	199 (22)		161 (35)	136 (28)	
Absent	530 (73)	712 (78)		304 (65)	353 (72)	
*FLT3*-TKD, *n* (%)			0.31			0.70
Present	89 (12)	100 (10)		60 (13)	59 (12)	
Absent	653 (88)	867 (90)		405 (87)	430 (88)	
*GATA1*, *n* (%)			0.43			1.00
Mutated	1 (<1)	0 (0)		0 (0)	1 (<1)	
Wild-type	679 (99)	895 (100)		465 (100)	488 (99)	
*GATA2*, *n* (%)			0.25			0.87
Mutated	29 (4)	50 (5)		18 (4)	20 (4)	
Wild-type	720 (96)	927 (95)		447 (96)	469 (96)	
*HNRNPK*, *n* (%)			1.00			1.00
Mutated	6 (1)	9 (1)		7 (2)	7 (1)	
Wild-type	743 (99)	968 (99)		458 (98)	482 (99)	
*IDH1*, *n* (%)			0.83			1.00
Mutated	104 (14)	132 (14)		41 (9)	44 (9)	
Wild-type	645 (86)	844 (86)		424 (91)	445 (91)	
*IDH2*, *n* (%)			0.66			0.36
Mutated	91 (12)	126 (13)		63 (14)	77 (16)	
Wild-type	658 (88)	851 (87)		402 (86)	412 (84)	
*JAK1*, *n* (%)			0.48			1.00
Mutated	6 (1)	12 (1)		2 (<1)	2 (<1)	
Wild-type	743 (99)	965 (99)		463 (99)	487 (99)	
*JAK2*, *n* (%)			0.11			0.33
Mutated	5 (1)	15 (2)		6 (1)	3 (1)	
Wild-type	722 (99)	931 (98)		459 (99)	486 (99)	
*JAK3*, *n* (%)			1.00			0.68
Mutated	8 (1)	11 (1)		3 (1)	2 (<1)	
Wild-type	741 (99)	966 (99)		462 (99)	487 (99)	
*KIT*, *n* (%)			0.05			0.39
Mutated	35 (5)	68 (7)		20 (4)	30 (6)	
Wild-type	698 (95)	890 (93)		451 (97)	469 (96)	
*KRAS*, *n* (%)			0.55			0.58
Mutated	52 (7)	60 (6)		25 (5)	31 (6)	
Wild-type	696 (93)	916 (94)		440 (95)	458 (94)	
*NF1*, *n* (%)			0.70			0.25
Mutated	26 (6)	37 (7)		0 (0)	3 (1)	
Wild-type	420 (94)	528 (93)		465 (100)	486 (99)	
*NOTCH1*, *n* (%)			0.70			0.34
Mutated	13 (2)	14 (2)		3 (1)	7 (1)	
Wild-type	668 (98)	881 (98)		462 (99)	482 (99)	
*NPM1*, *n* (%)			<0.001			<0.001
Mutated	305 (41)	276 (29)		247 (53)	153 (31)	
Wild-type	441 (59)	692 (71)		218 (47)	336 (69)	
*NRAS*, *n* (%)			0.90			0.12
Mutated	152 (20)	195 (20)		79 (17)	103 (21)	
Wild-type	597 (80)	782 (80)		386 (83)	386 (79)	
*PHF6*, *n* (%)			0.11			0.71
Mutated	20 (3)	40 (4)		13 (3)	16 (3)	
Wild-type	729 (97)	937 (96)		452 (97)	473 (97)	
*PTEN*, *n* (%)			1.00			0.49
Mutated	5 (1)	6 (1)		1 (<1)	0 (0)	
Wild-type	744 (99)	971 (99)		464 (99)	489 (100)	
*PTPN11*, *n* (%)			0.09			1.00
Mutated	85 (11)	86 (9)		46 (10)	49 (10)	
Wild-type	664 (89)	891 (91)		419 (90)	440 (90)	
*RAD21*, *n* (%)			1.00			1.00
Mutated	18 (2)	24 (2)		24 (5)	25 (5)	
Wild-type	731 (98)	953 (98)		441 (95)	464 (95)	
*RUNX1*, *n* (%)			0.04			<0.001
Mutated	69 (9)	122 (12)		46 (10)	88 (18)	
Wild-type	680 (91)	855 (88)		419 (90)	401 (82)	
*SETBP1*, *n* (%)			0.87			0.37
Mutated	15 (2)	22 (2)		1 (<1)	4 (1)	
Wild-type	734 (98)	955 (98)		464 (99)	485 (99)	
*SF1*, *n* (%)			0.45			--
Mutated	8 (1)	7 (1)		0 (0)	0 (0)	
Wild-type	741 (99)	970 (99)		465 (100)	489 (100)	
*SF3A1*, *n* (%)			1.00			0.62
Mutated	7 (1)	9 (1)		2 (<1)	1 (<1)	
Wild-type	742 (99)	968 (99)		463 (99)	488 (99)	
*SF3B1*, *n* (%)			0.20			0.28
Mutated	23 (3)	42 (4)		12 (3)	19 (4)	
Wild-type	724 (97)	935 (96)		453 (97)	470 (96)	
*SMC1A*, *n* (%)			0.54			0.003
Mutated	27 (4)	42 (4)		7 (2)	24 (5)	
Wild-type	722 (96)	935 (96)		458 (98)	465 (95)	
*SMC3*, *n* (%)			0.58			0.87
Mutated	25 (3)	28 (3)		20 (4)	20 (4)	
Wild-type	724 (97)	949 (97)		445 (96)	469 (96)	
*SRSF2*, *n* (%)			<0.001			<0.001
Mutated	45 (6)	113 (12)		23 (5)	79 (16)	
Wild-type	698 (94)	858 (88)		442 (95)	410 (84)	
*STAG2*, *n* (%)			0.19			<0.001
Mutated	21 (3)	39 (4)		20 (4)	50 (10)	
Wild-type	728 (97)	938 (96)		445 (96)	439 (90)	
*TET2*, *n* (%)			0.31			0.67
Mutated	122 (16)	141 (14)		80 (17)	79 (16)	
Wild-type	627 (84)	836 (86)		385 (83)	410 (84)	
*TP53*, *n* (%)			0.16			0.21
Mutated	53 (7)	88 (9)		38 (8)	29 (6)	
Wild-type	696 (93)	889 (91)		427 (92)	460 (94)	
*U2AF1*, *n* (%)			0.001			0.004
Mutated	18 (2)	54 (6)		5 (1)	20 (4)	
Wild-type	731 (98)	923 (94)		460 (99)	469 (96)	
*U2AF2*, *n* (%)			0.43			1.00
Mutated	1 (<1)	0 (0)		1 (<1)	2 (<1)	
Wild-type	679 (99)	895 (100)		464 (99)	487 (99)	
*WT1*, *n* (%)			0.02			0.92
Mutated	73 (10)	64 (7)		60 (13)	62 (13)	
Wild-type	676 (90)	913 (93)		405 (87)	427 (87)	

*P values for categorical variables are from Fisher’s exact test, *P* values for continuous variables are from the Wilcoxon rank-sum test.

**Table 2:** Frequencies of gene mutations arranged in functional groups in female and male patients with AML treated on the CALGB/Alliance frontline protocols and those treated on the German AML Cooperative Group frontline protocols.

Functional group^a^	CALGB/Alliance patients			AMLCG patients		
	Females *n* = 749	Males *n* = 977	P*	Females *n* = 465	Males *n* = 489	P*
Chromatin remodeling, *n* (%)			0.02			<0.001
Mutated	134 (18)	221 (23)		52 (11)	129 (26)	
Wild-type	615 (82)	756 (77)		413 (89)	360 (74)	
Cohesin complex, *n* (%)			0.31			<0.001
Mutated	87 (12)	130 (13)		65 (14)	115 (24)	
Wild-type	662 (88)	847 (87)		400 (86)	374 (76)	
Kinases, *n* (%)			0.08			0.08
Mutated	314 (44)	358 (39)		210 (45)	193 (39)	
Wild-type	404 (56)	552 (61)		255 (55)	296 (61)	
Methylation-related, *n* (%)			0.02			0.007
Mutated	385 (51)	446 (46)		288 (62)	260 (53)	
Wild-type	364 (49)	531 (54)		177 (38)	229 (47)	
*RAS* pathway, *n* (%)			0.13			0.10
Mutated	263 (35)	309 (32)		138 (30)	170 (35)	
Wild-type	486 (65)	668 (68)		327 (70)	319 (65)	
Spliceosome, *n* (%)			<0.001			<0.001
Mutated	82 (11)	210 (22)		40 (9)	129 (26)	
Wild-type	662 (83)	761 (78)		425 (91)	360 (74)	
Transcription factors, *n* (%)			0.06			0.001
Mutated	159 (24)	240 (28)		82 (18)	130 (27)	
Wild-type	517 (76)	625 (72)		383 (82)	359 (73)	
Tumor suppressors, *n* (%)			1.00			0.64
Mutated	141 (19)	184 (19)		97 (21)	89 (18)	
Wild-type	608 (81)	793 (81)		368 (79)	400 (82)	
Myelodysplasia-related, n (%)^b^			<0.001			<0.001
Mutated	207 (27)	358 (37)		107 (23)	208 (43)	
Wild-type	538 (72)	616 (63)		358 (77)	281 (57)	

^*^P values for categorical variables are from Fisher’s exact test, *P* values for continuous variables are from the Wilcoxon rank-sum test.

^a^Chromatin remodeling is mutated if one of *ASXL1*, *BCOR*, or *EZH2* is mutated. Kinases is mutated if one of *FLT3*-ITD, *FLT3*-TKD, or *KIT* is mutated. Methylation is mutated if one of *DNMT3A*, *IDH1*/*2*, or *TET2* is mutated. *RAS* pathway is mutated if one of *CBL*, *KRAS*, or *NRAS* is mutated. Spliceosome is mutated if one of *SF3B1*, *SRSF2*, *U2AF1* or *ZRSR2* is mutated. Transcription is mutated if one of *CEBPA*, *ETV6*, *IKZF1*, *GATA2* or *RUNX1* is mutated. Tumor suppressor is mutated if *PHF6*, *TP53* or *WT1* is mutated.

^b^The category “myelodysplasia-related” mutations has been included in the Table because it is used as an adverse-risk criterion in the widely adopted 2022 European LeukemiaNet genetic-risk classification [1, 44]. This category contains mutations in the *SRSF2*, *SF3B1*, *RUNX1*, *U2AF1*, *ZRSR2, ASXL1*, *EZH2, BCOR* and *STAG2* genes.

To validate the observed sex-associated differences, we compared the frequencies of cytogenetic findings (Supplementary Table 1) and gene mutations in patients from AMLCG (Tables 1 and 2). The results were largely concordant, with CN-AML and all mutations, except *KIT* and *WT1* mutations, which differed between males and females among CALGB/Alliance patients being also significantly different in the AMLCG cohort. However, in the latter patient population, females also less often carried *EZH2* (*P* = 0.005), *SMC1A* (*P* = 0.003) and *STAG2* (*P* < 0.001) mutations and mutations in the cohesion complex genes (*P* < 0.001) than males.

The proportions of patients assigned to genetic-risk groups in the 2022 ELN classification [1] also differed between sexes. In the CALGB/Alliance cohort, a higher percentage of female patients was categorized in the intermediate-risk group (30% vs 20%) and a lower percentage of women was included in the adverse-risk group (31% vs 44%, Supplementary Table 1).

### Sex-associated differences in co-occurring molecular alterations

**Fig. 1:**
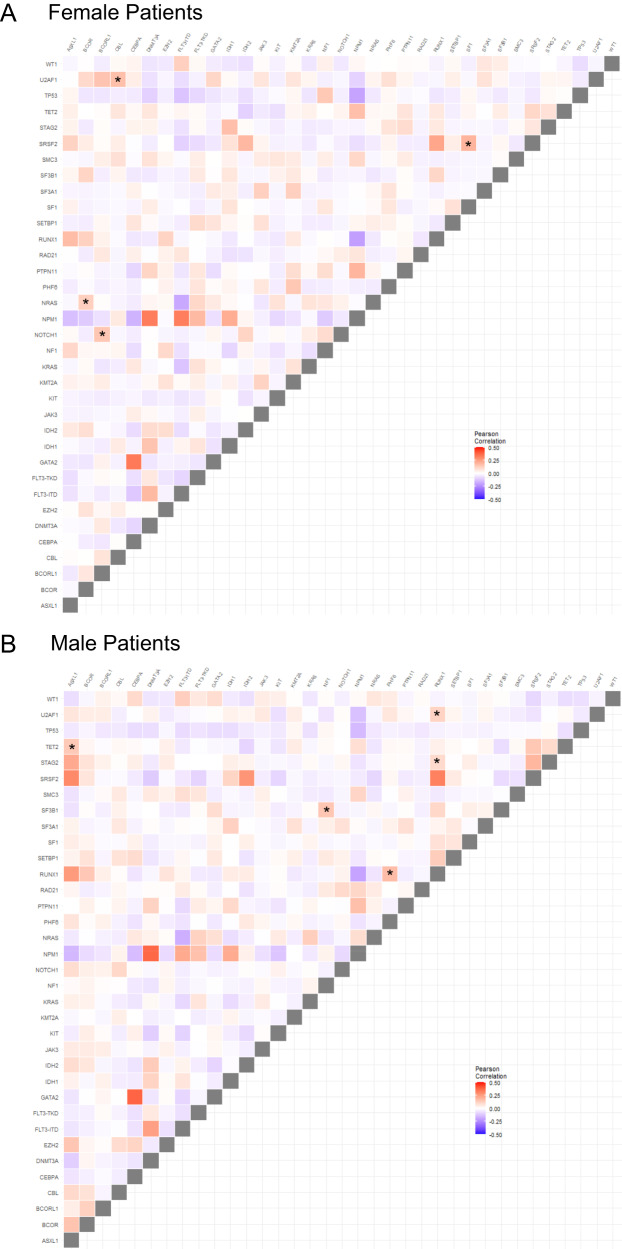
Differences in co-existing molecular features between female and male patients with AML. Triangle correlation plot depicting co-occurring mutations in **A** female and **B** male patients with AML treated on CALGB/Alliance frontline protocols. All squares denoting gene mutation pairs that differ between the sexes are marked using stars.

We next compared patterns of co-occurring recurrent mutations in male and female patients and differences between sexes (Pearson correlation <0.2 and *P* < 0.0002 for one sex and Pearson correlation >0.03 and *P* > 0.5). We identified four gene mutation pairs, including *NRAS*/*BCOR*, *U2AF1*/*CBL*, *NOTCH1*/*BCORL1*, and *SF1*/*SRSF2* whose presence was strongly associated with female patients. Conversely, *ASXL1*/*TET2*, *NF1*/*SF3B1*, *RUNX1*/*PHF6*, *RUNX1*/*U2AF1* and *RUNX1*/*STAG2* were strongly positively associated with male patients. Conversely, *WT1*/*SRSF2* tended to be mutually exclusive in males but less so in females (Fig. 1).

### Treatment outcome of adults with AML aged <60 years with respect to patient sex

We analyzed clinical outcomes of 381 female and 463 male AML patients aged 17–59 years in the CALGB/Alliance cohort, for whom frontline treatment with intensive induction chemotherapy is currently still a standard of care. We found no significant differences in CR and early death rates, DFS or OS between female and male AML patients (Supplementary Table 2). There were also no differences between sexes in CR and early death rates or DFS in the AMLCG cohort. However, male German patients aged 18–59 years had a shorter OS than female patients (5-year rates, 42% vs 51%, *P* = 0.005).

### Sex-associated prognostic impact of genetic alterations in patients aged < 60 years

Since cytogenetic and molecular genetic findings are routinely used for risk stratification of AML patients, we assessed sex-specific outcomes based on the 2022 ELN genetic-risk categories [1]. Despite differences in proportions of females and males in the intermediate- and adverse-risk groups, DFS and OS was essentially equal within each genetic-risk group, except for longer OS of female adverse-risk patients (*P* < 0.001; Supplementary Fig. 1A, B).

**Fig. 2:**
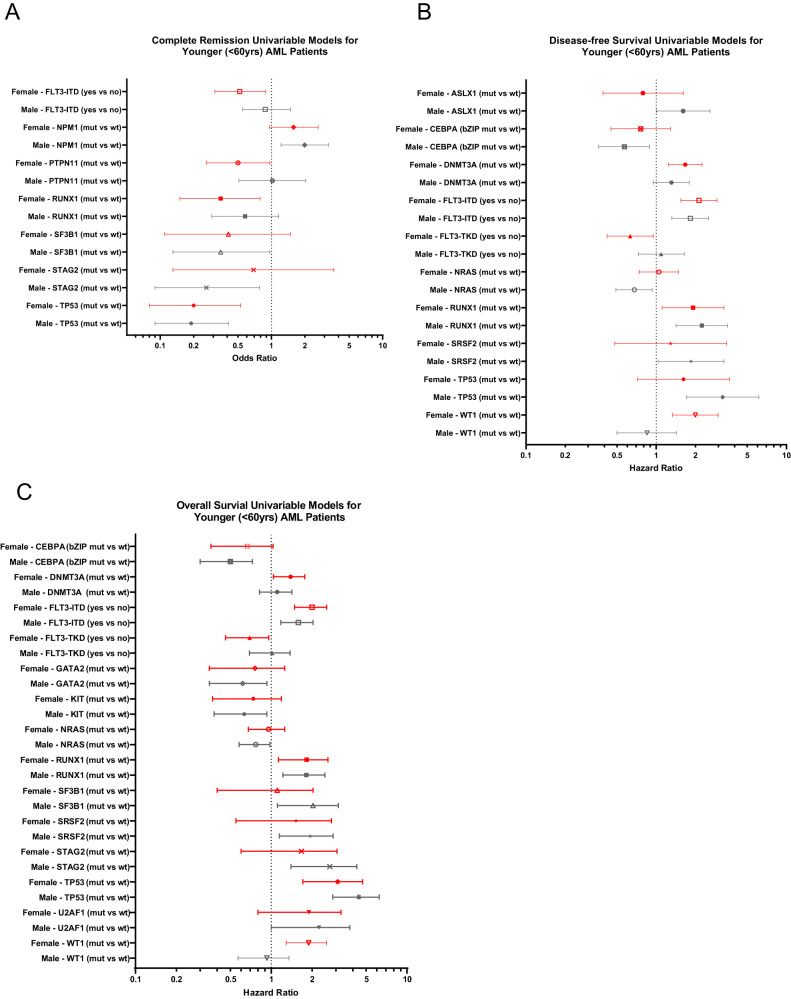
Outcomes of patients with AML aged <60 years who were treated on the CALGB/Alliance study protocols. Forest plot illustrating univariable analyses of **A** complete remission, **B** disease-free survival and **C** overall survival. Depicted in the plot are all gene mutations with sex-specific survival impact for either male or female patients in univariable analyses for the respective outcome endpoint. Results of the corresponding multivariable analyses and markers with significance are shown in Supplementary Table 3.

Next, we assessed associations of recurrent genetic alterations with outcome of CALGB/Alliance patients. In univariable analysis (UVA) for CR, presence of *FLT3*-ITD, *PTPN11* and *RUNX1* mutations affected outcome of only female patients (Fig. 2A), of which *FLT3*-ITD and *PTPN11* mutations remained significantly associated with CR achievement also in subsequent multivariable analysis (MVA, Supplementary Table 3). In contrast, a normal karyotype and *SF3B1* mutations were adverse predictors only in male patients in both UVA and MVA.

For DFS in female patients, *DNMT3A* and *WT1* mutations associated with shorter, and *FLT3*-TKD mutations with longer DFS in both UVA and MVA (Fig. 2B, Supplementary Fig. 2A). While for male patients detection of *ASXL1*, *CEBPA*^bZIP^, *NRAS*, *SRSF*2 and *TP53* mutations had sex-specific survival impact in UVA, only *CEBPA*^bZIP^ mutations were significant in multivariable models.

**Fig. 3:**
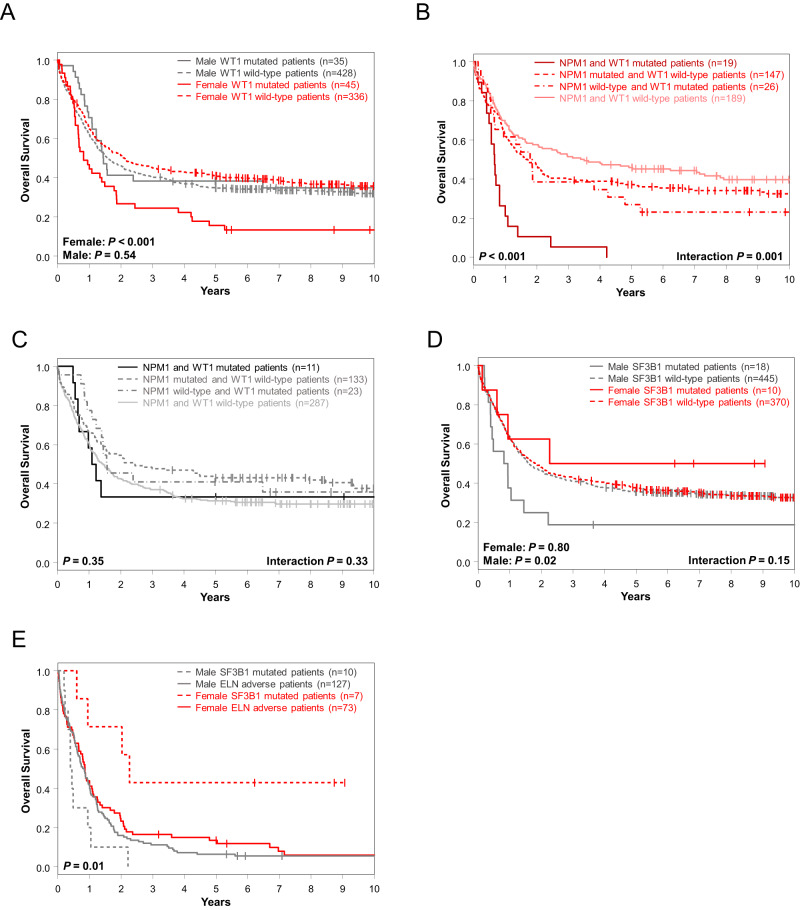
Overall survival of female and male patients with AML according to their *WT1*, *NPM1* and *SF3B1* mutation status. **A** Overall survival of female and male patients with and without *WT1* mutations and overall survival of **B** female and **C** male patients according to their *NPM1* and *WT1* mutation status. **D** Overall survival of female and male patients with and without *SF3B1* mutations. **E** Overall survival of female and male patients classified in the 2022 ELN adverse-risk group because of the presence of *SF3B1* mutation (and lack of favorable-risk genetic markers) and of the remaining female and male patients classified in the 2022 ELN adverse-risk group who did not have *SF3B1* mutation.

In univariable analysis of OS, *FLT3*-TKD, *DNMT3A* and *WT1* mutations affected outcome of only female patients (Fig. 2C) of which, again, *WT1* mutations held their significance also in multivariable analyses (Fig. 3A). Notably, the adverse outcome impact of female *WT1*-mutated patients seemed to be driven by *WT1/NPM1* co-mutated female patients (Fig. 3B, C). OS of men only was influenced by *CEBPA*^bZIP^, *GATA2*, *KIT*, *NRAS*, *SF3B1*, *SRSF2*, *STAG2* and *U2AF1* mutations in UVA, of which *SF3B1* mutations had again sex-specific survival association in MVA, too (Fig. 3D, Supplementary Table 3).

Focusing on those sex-specific molecular alterations that held their significance in both UV and MV models and at least 2 survival endpoints (female: *WT1* mutations [DFS, OS]; male: *SF3B1* mutations [CR, OS]), we tested their outcome impacts in the AMLCG cohort.

While *WT1* mutations associated with a trend for inferior DFS in female AMLCG patients, there was no association with inferior OS, possibly suggestive of a rescue effect of more frequently used intensive consolidation including allogeneic HSCT which is more commonly administered in Germany (Supplementary Fig. 2B, C). Although limited by relatively small sample sizes, *SF3B1*-mutated male patients in the AMLCG cohort also had a lower CR rate (38% vs 72%, *P* = 0.04) and tended to have inferior OS (Supplementary Fig. 3).

As *SF3B1* mutations demonstrated distinct prognostic differences and were recently added to the 2022 ELN classification as adverse-risk outcome prognosticator (in the absence of favorable-risk genetic markers), we compared OS of adverse-risk female and male patients with *SF3B1* mutations and OS of the remaining adverse-risk female and male patients (i.e., without *SF3B1* mutations). While male *SF3B1*-mutated 2022 ELN adverse-risk patients and both female and male adverse-risk patients without *SF3B1* mutations had similarly poor overall survival, female *SF3B1*-mutated adverse-risk patients had significantly longer OS (5-year rates, 43% vs 8%, *P* = 0.01) than other 2022 ELN adverse-risk patients (Fig. 3E).

### Treatment outcome of adults with AML aged ≥60 years with respect to patient sex and the sex-associated prognostic impact of genetic alterations

Given the generally poor survival of older patients with AML, we performed a subset outcome analyses of the AML patients aged 60 years and older included in our study. We did not find significant differences between sexes among 524 older patients treated on the CALGB/Alliance protocols nor among 414 older German patients (Supplementary Table 4). There were no significant differences in DFS or OS between female and male patients in any of the 2022 ELN genetic-risk groups, with patients in the intermediate-risk and adverse-risk groups performing similarly poorly (Supplementary Fig. 4A, B). Although drawing definitive conclusions from the multivariable analyses is difficult because of the generally poor treatment outcome of older patients with AML, we found that such mutations included in the 2022 ELN genetic-risk classification as *FLT3*-ITD, *NPM1* and *TP53* mutations were among main factors affecting CR rates and survival (Supplementary Table 5). Only in men, DFS was also negatively affected by *PTPN11* mutations and OS by *KRAS* mutations.

### Sex-specific alternative splicing events and lineage-associated gene expression patterns

To investigate the potential role of sex bias on gene and splicing programs, we next analyzed patients with existing RNAseq data (*n* = 848) to quantify alternative splicing events (ASE) and differentially expressed genes (DEGs) associated with recurrent gene mutations or cytogenetic findings using the AltAnalyze workflow, including those affecting spliceosome genes and other recurrent AML-associated genes or cytogenetic features (*n* = 24) and select clinical/patient demographic variables.

**Fig. 4:**
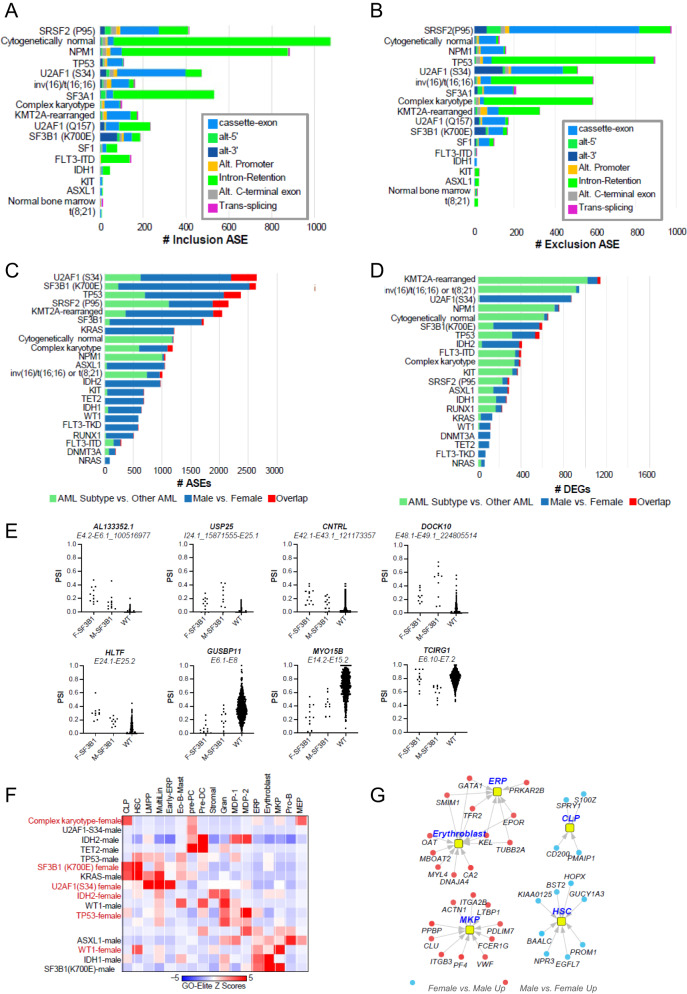
Alternative splicing events (ASEs) segregated by mechanism of predicted regulation for recurrent mutations, gene fusions and clinical covariates in the CALGB/Alliance RNA-Seq cohort. The extent of exon/intron **A** inclusion or **B** exclusion ASEs. The extent of unique sex-associated ASEs **C** or differentially expressed genes (DEGs) **D** associated with each patient subtype, unique regulated events or genes compared with all other AMLs and sex/subtype overlapping events/genes, derived from the software AltAnalyze. Green color denotes the number of unique ASEs or DEGs in all patients with the indicated molecular genetic or cytogenetic subtype versus all other AML patients in the cohort. Blue indicates the number of unique ASEs or DEGs in males versus females in the indicated AML subtype and red illustrates overlap between subtype-associated ASEs or DEGs and male versus female (subtype regulated events/genes that vary according to patient sex). **E** Quantification of *SF3B1*-mutated sex-associated alternative splicing events as percent spliced in (PSI) values for the indicated parental exon-exon junction. F = female, M = Male. **F** Heatmap of gene set enrichments (GO-Elite) for known AML subtypes against human bone marrow cell-type markers to identify lineage skewing, BM cell-type markers were derived from extensive prior human single-cell analyses [43], to identify markers of rare hemopoietic stem cells, progenitors, and immune cell-types. Only AML subtype sex-associated gene sets with significant enrichments are shown. **G** Sex-associated differentially expressed genes in *SF3B1*-mutated males versus females corresponding to human bone marrow progenitor populations.

To set the stage, we first determined the spectrum of mutations, which result in common splicing impacts via supervised and unsupervised analyses irrespective of sex, which could be verified in independent AML cohorts (Supplementary Methods and Supplementary Fig. 5A, B). Most AML-associated mutations and chromosome rearrangements resulted in splicing impacts, most significantly associated with splicing-factor mutations (*SRSF2*-P95*, *U2AF1*-S34*, *SF3B1*, *SF3A1*, *U2AF1*-Q157*, *SF1*), common mutations (*NPM1*, *TP53*), CN-AML, complex karyotype, and chromosome rearrangements resulting in gene fusions involving *CBFB* and *KMT2A* (Fig. 4A, B).

Next, we compared female and male patients in each of these defined subtypes. We considered subtypes with >17 samples and an unadjusted *P*-value for these comparisons. Interestingly, we found different magnitudes of splicing and gene-expression impacts associated with sex in AML subtypes, with generally greater impacts associated with ASEs versus DEGs (Fig. 4C, D). In general, splicing-factor mutations (*SRSF2*-P95*, *U2AF1-*S34*, *SF3B1*, *SF3A1*, *SF3A1*, *SF1*) resulted in large sex-associated differences for both ASEs and DEGs. Notably, while it has been extensively documented that *SF3B1*-K700E and functionally similar mutations result in alternative splice sites only selected for within the presence of these mutations, examination of *SF3B1-*K700E and related mutation ASEs predicted sex bias for several of previously annotated (*USP25, MYO15B*) and novel *SF3B1*-K700E selected cryptic splice sites (*AL133352.1, CNTRL, DOCK10*), in addition to non-cryptic splice sites with clear sex bias (Fig. 4E). At the pathway level, we found that *SF3B1* mutations in male patients transcriptionally induce genes associated with integrin cell-surface receptor-linked signaling pathways (MAP kinase, Netrin, PECAM1, Ephrin B, interferon alpha/beta signaling, L1CAM), whereas *RUNX1-* and *TP53*-mutated male patients induce inflammatory response pathways (IL12, FGF, CXCR4 and TCR signaling). Further, complex karyotype and *TP53* mutations in female patients induce a distinct set of inflammatory and immune signaling programs (Supplementary Fig. 5C, D). When we considered marker genes for hematopoietic human progenitor and differentiated cell populations, we noted distinct lineage enrichments shared by different sets of mutations. In particular, *SF3B1* (females) and *KRAS* (males) were enriched in hematopoietic stem cells, MultiLin, common lymphoid precursor and eosinophil/mast programs, whereas *SF3B1* (males) and *WT1* (females) induced a dominant megakaryocytic progenitors/erythroid progenitors (Fig. 4F). We note that in male *SF3B1-*mutated patients, genes associated with these programs represent well-defined lineage determining factors, such as *HOPX* and *PROM1* for hematopoietic stem cells, *ITGA2B*, *VWF* and *PF4* for megakaryocytic progenitors and *EPOR* and *GATA1* for erythroid progenitors (Fig. 4G). Together, these data indicate that sex contributes to both alternative splicing and transcriptional differences among patients with splicing factor and other dominant AML mutations.

## Discussion

The discovery of recurrent cytogenetic and molecular genetic alterations has improved our understanding of AML biology and resulted in the routine use of pretreatment genetic alterations for risk stratification [1–19]. However, genetic changes should not be assessed in isolation, because their effects may be influenced by such factors as patients age [1, 2, 9, 20] and/or racial-ethnic identity [21, 22]. Whereas female and male patients with AML share the vast majority of genetic information, except for at least 26 protein-coding genes located on the Y chromosome, large-scale genomic studies of health and disease have revealed profound differences in sex-biased gene-regulatory networks [31] and splicing events [32] contributing to phenotypic sex differences.

Recently, De-Morgan et al. [23] also demonstrated specific preleukemic genes that were more frequently mutated in men than women with AML. Consistent with previous reports, our study found a female predominance of such most common (de novo) AML-associated mutations as *NPM1*, *DNMT3A* and *FLT3*-ITD, and a male predominance of spliceosome complex gene mutations and other myelodysplasia-related genes, many of which are found on the X-chromosome (*BCOR*, *BCORL1*, *SMC1A*, *STAG2*, *ZRSR2*). These findings might be interesting in the context of the known higher incidence of AML in males compared to females, and suggestive of sex-specific differences in the disease biology. Interestingly, analyzing gene expression and alternative splicing patterns first on a global scale followed by sex-specific analyses, we identified differential splicing events that associated with sex, as well as cell lineage, depending on the gene mutation. Surprisingly, our data suggest that shared pathway-level impacts for pairs of mutations exist, which tend to be associated with sex, whereas cell-type associated impacts indicate a lineage bias in programs, which often differ from the pathway. Although biologic reasons for the male-specific outcome association of *SF3B1* mutations are still unclear, the differential effects on cell lineages suggested by GSEA indicate that sex-specific cell programs influencing leukemogenesis and leukemic cell responses may exist.

We detected male-specific association of *SF3B1* mutations with higher resistance to induction chemotherapy and shorter overall survival. Notably, *SF3B1* mutations, in the absence of favorable-risk genetic markers, have recently been added as an adverse-risk prognosticator to the 2022 ELN genetic-risk classification [1]. Our data expand upon the work of the GenoMed4All consortium and Maggioni et al. [33], which also demonstrated sex-specific biases at the single-gene level in myelodysplastic syndromes (MDS) and a male specificity for co-mutational pathways in splicing-related genes. Our work, if confirmed, ultimately suggest that *SF3B1* mutations might constitute a sex-specific prognosticator, which could be taken into account in the future revisions of the ELN genetic-risk classification.

The negative prognostic impact of *WT1* mutations has been repeatedly reported in the past [34–39], but their association with female sex have hitherto not been recognized. In our study, an association of *WT1* mutations with women was seen only in the CALGB/Alliance cohort, but was not observed in the AMLCG patients. As patients treated in Europe receive allogeneic HSCT in first CR more often, it is possible that more intensive consolidation might alleviate the adverse prognostic impact of *WT1* mutations. This discrepancy might warrant further investigation in prospective studies, to help determine the appropriate consolidation strategy for female patients carrying *WT1* mutations. Moreover, adverse outcome of *WT1*-mutated women seems to be associated with co-existence of *NPM1* mutations, which was previously suggested to confer high treatment resistance and poor survival rates [17, 40].

Limitations of our study include the fact that we studied only patients with de novo AML, and that the Alliance patients did not undergo allogeneic HSCT in first CR and were almost exclusively treated with intensive induction chemotherapy followed by consolidation therapy. Hence, future studies are needed to assess associations between sex and molecular features and survival in patients undergoing allogeneic HSCT and in those receiving emerging targeted therapies, which is particularly important in older patients. Furthermore, we detected sex-associated differences in the frequencies of myelodysplasia-related gene mutations among patients diagnosed with de novo AML, not in patients with AML evolving from an antecedent MDS whom we did not analyze. It will be thus important to perform dedicated analyses in patients with secondary AML evolving from MDS, especially since sex disparities with respect to genotypes, phenotypes and outcomes have previously been reported in patients diagnosed with MDS [41, 42].

In summary, our study assessed potential sex-associated biologic and prognostic implications of genetic alterations in large patient cohorts. Although most established genetic alterations affected outcomes of both sexes, select gene mutations were associated with patient sex. This was likely missed previously because of routinely performed sex-pooled outcome analyses. We identified *SF3B1* mutations as a male-specific and *WT1* mutations as female-specific prognosticators of poor survival in patients treated with conventional chemotherapy. Differences in the frequencies of AML-associated genetic alterations and mutation-specific differential splicing events, with possible subsequent phenotypic changes, highlight the importance of considering the patients’ sex in analyses examining leukemia biology and prognostic significance of genetic alterations and their role in clinical decision-making in AML.

### Supplementary information

Supplementary Information

## Data Availability

Patient data used in survival analyses were obtained from the Alliance Statistics and Data Management Center. Individual participant data will not be shared.
