# Supplementary material for: Sex-associated differences in frequencies and prognostic impact of recurrent genetic alterations in adult acute myeloid leukemia (Alliance, AMLCG)
Source: Leukemia. 2023 Nov 28;38(1):45–57. doi: 10.1038/s41375-023-02068-8 (PMC10776397; doi:10.1038/s41375-023-02068-8)

**Supplementary Information**

**Sex-associated differences in frequencies and prognostic impact of recurrent genetic alterations in adult acute myeloid leukemia (Alliance, AMLCG)**

Michael Ozga,^1^ Deedra Nicolet,^2,3^ Krzysztof Mrózek,^1,2,^* Ayse S. Yilmaz,^1,2^

Jessica Kohlschmidt,^2,3^ Karilyn T. Larkin,^1,2^ James S. Blachly,^1,2^ Christopher C. Oakes,^1,2^

Jill Buss,^1,2^ Christopher J. Walker,^1,2^ Shelley Orwick,^1^ Vindi Jurinovic,^4^

Maja Rothenberg-Thurley,^4^ Annika Dufour,^4^ Stephanie Schneider,^4,5^ Maria Cristina Sauerland,^6^ Dennis Görlich,^6^ Utz Krug,^7^ Wolfgang E. Berdel,^8^ Bernhard J. Woermann,^9^

Wolfgang Hiddemann,^4,10^ Jan Braess,^11^ Marion Subklewe,^4,10,12^ Karsten Spiekermann,^4,10,12^ Andrew J. Carroll,^13^ William G. Blum,^14^ Bayard L. Powell,^15^ Jonathan E. Kolitz,^16^

Joseph O. Moore,^17^ Robert J. Mayer,^18^ Richard A. Larson,^19^ Geoffrey L. Uy,^20^ Wendy Stock,^19^ Klaus H. Metzeler,^21^ H. Leighton Grimes,^22,23^ John C. Byrd,^24^ Nathan Salomonis,^23,25,†^

Tobias Herold,^4,10,12,†^ Alice S. Mims^1,2,†^ and Ann-Kathrin Eisfeld^1,2,^*^,†^

^1^The Ohio State University Comprehensive Cancer Center, Columbus, OH, USA.

^2^The Ohio State University Comprehensive Cancer Center, Clara D. Bloomfield Center for Leukemia Outcomes Research, Columbus, OH, USA.

^3^Alliance Statistics and Data Management Center, The Ohio State University Comprehensive Cancer Center, Columbus, OH, USA.

^4^Laboratory for Leukemia Diagnostics, Department of Medicine III, University Hospital, LMU Munich, Munich, Germany.

^5^Institute of Human Genetics, University Hospital, LMU Munich, Munich, Germany.

^6^Institute of Biostatistics and Clinical Research, University of Münster, Münster, Germany.

^7^Department of Medicine 3, Klinikum Leverkusen, Leverkusen, Germany.

^8^Department of Medicine, Hematology and Oncology, University of Münster, Münster, Germany.

^9^German Society of Hematology and Oncology, Berlin, Germany.

^10^German Cancer Research Center (DKFZ), Heidelberg, Germany.

^11^Department of Oncology and Hematology, Hospital Barmherzige Brüder, Regensburg, Germany.

^12^German Cancer Consortium (DKTK), Partner Site Munich, Munich, Germany.

^13^Department of Genetics, University of Alabama at Birmingham, Birmingham, AL, USA.

^14^Emory University School of Medicine, Atlanta, GA, USA.

^15^ Wake Forest University Health Sciences, Winston-Salem, NC, USA.

^16^Monter Cancer Center, Hofstra Northwell School of Medicine, Lake Success, NY, USA.

^17^Duke Cancer Institute, Duke University Health System, Durham, NC, USA.

^18^Department of Medical Oncology, Dana-Farber/Partners CancerCare, Boston, MA, USA.

^19^University of Chicago Medical Center, Chicago, IL, USA.

^20^Division of Oncology, Washington University School of Medicine, St. Louis, MO, USA.

^21^Department of Hematology, Cellular Therapy, and Hemostaseology, Leipzig University Hospital, Leipzig, Germany.

^22^Division of Immunobiology, Cincinnati Children’s Hospital, University of Cincinnati, Cincinnati, OH, USA.

^23^Department of Pediatrics, University of Cincinnati, Cincinnati, OH, USA.

^24^Department of Internal Medicine, University of Cincinnati, Cincinnati, OH, USA.

^25^Division of Biomedical Informatics, Cincinnati Children’s Hospital, University of Cincinnati, Cincinnati, OH, USA.

* **Correspondence:** Dr. Ann-Kathrin Eisfeld, The Ohio State University Comprehensive Cancer Center, 460 West 12^th^ Avenue, Room 850, Columbus, OH 43210-1228, USA, phone: 614-477-5667, e-mail: [Ann.Kathrin.Eisfeld@osumc.edu](mailto:Ann.Kathrin.Eisfeld@osumc.edu) or Dr. Krzysztof Mrózek, The Ohio State University Comprehensive Cancer Center, OSU Medical Center, Room 351, 650 Ackerman Rd, Columbus, OH 43202, USA; e-mail: [krzysztof.mrozek@osumc.edu](mailto:krzysztof.mrozek@osumc.edu).

^†^ Nathan Salomonis, Tobias Herold, Alice S. Mims and Ann-Kathrin Eisfeld contributed equally to this work as senior authors.

**Participating institutions**

The following Cancer and Leukemia Group B (CALGB)/Alliance for Clinical Trials in Oncology (Alliance) institutions participated in this study and contributed at least five patients. For each of these institutions, the current or last principal investigator and the cytogeneticists who analyzed the cases are listed as follows:

The Ohio State University Medical Center, Columbus, OH: Claire F. Verschraegen, Karl S. Theil, Diane Minka and Nyla A. Heerema; North Shore University Hospital, Manhasset, NY: Jonathan E. Kolitz, Prasad R. K. Koduru, Ayala Aviram-Goldring and Chandrika Sreekantaiah; Wake Forest University School of Medicine, Winston-Salem, NC: Heidi D. Klepin, P. Nagesh Rao, Wendy L. Flejter and Mark Pettenati; Roswell Park Cancer Institute, Buffalo, NY: Ellis G. Levine and AnneMarie W. Block; Dana Farber Cancer Institute, Boston, MA: Harold J. Burstein, Ramana V. Tantravahi, Cynthia C. Morton and Paola Dal Cin; Washington University School of Medicine, St. Louis, MO: Nancy L. Bartlett, Michael S. Watson, Eric C. Crawford, Jaime Garcia-Heras, Peining Li and Shashikant Kulkarni; University of Iowa Hospitals, Iowa City, IA: Umar Farooq and Shivanand R. Patil; University of North Carolina, Chapel Hill, NC: Matthew I. Milowsky and Kathleen W. Rao; University of Chicago Medical Center, Chicago, IL: Hedy L. Kindler, Diane Roulston, Katrin M. Carlson, Yanming Zhang and Michelle M. LeBeau; Duke University Medical Center, Durham, NC: Jeffrey Crawford, Sandra H. Bigner, Mazin B. Qumsiyeh, John Eyre and Barbara K. Goodman; University of Maryland Greenebaum Cancer Center, Baltimore, MD: Heather D. Mannuel, Joseph R. Testa, Maimon M. Cohen, Judith Stamberg and Yi Ning; Dartmouth Medical School, Lebanon, NH: Konstantin H. Dragnev, Doris H. Wurster-Hill and Thuluvancheri K. Mohandas; Christiana Care Health Services, Inc., Newark, DE: Gregory A. Masters, Digamber S. Borgaonkar, Jeanne M. Meck, and Kathleen Richkind; Rhode Island Hospital, Providence, RI: Howard P. Safran, Teresita Padre-Mendoza, Hon Fong L. Mark, Shelly L. Kerman and Aurelia Meloni-Ehrig; Ft. Wayne Medical Oncology/Hematology, Ft. Wayne, IN: Sreenivasa Nattam and Patricia I. Bader; Weill Medical College of Cornell University, New York, NY: Scott Tagawa, Ram S. Verma, Prasad R.K. Koduru, Andrew J. Carroll and Susan Mathew; Western Pennsylvania Hospital, Pittsburgh, PA: Gene G. Finley and Gerard R. Diggans; Massachusetts General Hospital, Boston, MA: David Ryan, Leonard L. Atkins, Cynthia C. Morton and Paola Dal Cin; University of Vermont Cancer Center, Burlington, VT: Peter A. Kaufman, Elizabeth F. Allen and Mary Tang; SUNY Upstate Medical University, Syracuse, NY: Stephen L. Graziano, Larry Gordon and Constance K. Stein; University of Massachusetts Medical Center, Worcester, MA: William V. Walsh, Philip L. Townes, Vikram Jaswaney, Kathleen Richkind, Patricia Miron and Michael J. Mitchell; University of California San Diego Moores Cancer Center, San Diego, CA: Lyudmila A. Bazhenova, E. Robert Wassman, Jr., Renée Bernstein and Marie L. Dell'Aquila; Eastern Maine Medical Center, Bangor, ME: Sarah J. Sinclair and Laurent J. Beauregard; Long Island Jewish Medical Center, Lake Success, NY: Jonathan E. Kolitz, Alan L. Shanske, Prasad R. K. Koduru, Ayala Aviram-Goldring and Chandrika Sreekantaiah; Mount Sinai School of Medicine, New York, NY: Michael A. Schwartz and Vesna Najfeld; University of Alabama at Birmingham, Birmingham, AL. Robert Diasio and Andrew J. Carroll; University of Tennessee Cancer Center, Memphis, TN: Harvey B. Niell and Sugandhi A. Tharapel; Walter Reed National Military Medical Center, Bethesda, MD: Karen G. Zeman, Rawatmal B. Surana, Digamber S. Borgaonkar, Karl S. Theil and Kathleen E. Richkind; University of Missouri/Ellis Fischel Cancer Center, Columbia, MO: Puja Nistala, Jeffrey R. Sawyer, Tim Hui-Ming Huang and Linda M. Pasztor; University of Minnesota, Minneapolis, MN: Robert A. Kratzke, Diane C. Arthur and Betsy A. Hirsch; University of Illinois, Chicago, IL: John G. Quigley, Maureen M. McCorquodale, Kathleen E. Richkind and Valerie Lindgren; Virginia Commonwealth University, Richmond, VA: Zhijian Chen, Mary H. Hackney and Colleen Jackson-Cook; University of Puerto Rico, San Juan, Puerto Rico: Eileen I. Pacheco, Leonard L. Atkins, Cynthia C. Morton and Paola Dal Cin; University of Nebraska Medical Center, Omaha, NE: Apar Ganti and Warren G. Sanger; University of California at San Francisco, San Francisco, CA: Charalambos Andreadis and Kathleen E. Richkind; Georgetown University Medical Center, Washington, DC: Minnetta C. Liu and Jeanne M. Meck; Southern Nevada Cancer Research Foundation CCOP, Las Vegas, NV: John A. Ellerton and Marie L. Dell'Aquila.

# Patients and treatment

*CALGB/Alliance patients*

A total of 1,726 patients with untreated *de novo* acute myeloid leukemia (AML), enrolled on CALGB/Alliance study protocols detailed below, were included. CALGB is now part of the Alliance for Clinical Trials in Oncology. All patients provided written informed consent for participation in the treatment studies and in companion studies CALGB 8461 (cytogenetic studies; Trial Registration Number: NCT00048958), CALGB 9665 (leukemia tissue bank; NCT00899223) and CALGB 20202 (molecular studies; NCT00900224), which involved collection of pretreatment bone marrow (BM) and blood samples. All study protocols were in accordance with the Declaration of Helsinki and approved by Institutional Review Boards at each treatment center.

Patients were treated on CALGB/Alliance studies 19808 (n=348), 10503 (n=323), 9621 (n=181), 8525 (n=99), 9222 (n=108), 10603 (n=78), 8821 (n=9), 9022 (n=16), 9120 (n=2), 8721 (n=1), 9720 (n=224), 9420 (n=23), 8923 (n=31) 10201 (n=175), 10502 (n=37), 11002 (n=50) 11001 (n=17), 8361 (n=2), 8621 (n=1) and 10801 (n=1). Among younger patients, aged <60 years, those enrolled onto CALGB 19808 were randomly assigned to receive induction chemotherapy with cytarabine, daunorubicin, and etoposide with or without PSC-833 (valspodar), a multidrug resistance protein inhibitor.^1^ On achievement of complete remission (CR), patients were assigned to intensification with high-dose cytarabine and etoposide for stem-cell mobilization followed by myeloablative treatment with busulfan and etoposide supported by autologous peripheral blood HSCT. Patients enrolled on CALGB 10503 were assigned to receive induction chemotherapy consisting of cytarabine, daunorubicin, and etoposide. Upon achievement of CR, patients received high-dose cytarabine (HiDAC) and etoposide for stem-cell mobilization followed by myeloablative treatment with busulfan and etoposide supported by autologous peripheral HSCT. Patients not eligible for HSCT received HiDAC. After intensification, patients received the DNA methyltransferase inhibitor decitabine for maintenance.^2^ Patients enrolled on CALGB 9621 were treated similarly to those on CALGB 19808, as previously reported.^3^ Patients enrolled onto CALGB 8525 received induction chemotherapy with cytarabine and daunorubicin, and were randomly assigned to consolidation with different doses of cytarabine followed by maintenance treatment.^4^ Patients on protocol CALGB 9222 received induction chemotherapy consisting of cytarabine in combination with daunorubicin followed by consolidation with one cycle of HiDAC. Different doses of mitoxantrone were explored as well, and the consolidation treatment was randomized to three cycles of monotherapy with HiDAC or consolidation with one cycle of HiDAC, a cycle of cyclophosphamide and etoposide, and one cycle of mitoxantrone and diaziquone.^5^ In CALGB 10603, cytarabine and daunorubicin followed by consolidation with HiDAC was applied with or without midostaurin (PKC-412).^6^ Patients enrolled on CALGB 8821 received cytarabine combined with daunorubicin as induction and mitoxantrone/diaziquone, and etoposide/cyclophosphamide were then successively administered in two intensification courses.^7^ Patients enrolled onto CALGB 9022 received induction chemotherapy consisting of cytarabine in combination with daunorubicin followed by consolidation with one cycle of HiDAC, a cycle of cyclophosphamide and etoposide, and one cycle of mitoxantrone and diaziquone.^8^ Patients enrolled on CALGB 9120 received standard induction chemotherapy. After CR had been achieved, idarubicin (two days) and cytarabine (five days) were administered. Patients with histocompatible siblings were offered allogeneic marrow transplantation (HSCT), whereas the remaining patients were randomly assigned to receive a single course of high-dose cytarabine or transplantation of autologous marrow treated with perfosfamide (4-hydroperoxycyclophosphamide).^9^

Older patients, aged ≥60 years, enrolled onto CALGB 9720 and CALGB 9420 received induction chemotherapy consisting of cytarabine in combination with daunorubicin and etoposide, and were randomized to the arm with or without the multidrug resistance protein modulator valspodar (PSC 833). The PSC-833 arm was closed after random assignment of 120 patients due to a high number of early deaths.^10-12^ The patients received a single cytarabine/daunorubicin consolidation course and were randomly assigned to low-dose recombinant interleukin-2 maintenance therapy or none.^11,12^ Older patients enrolled onto CALGB 8923 received induction chemotherapy consisting of cytarabine in combination with daunorubicin, and were randomly assigned to receive postremission treatment with cytarabine alone or in combination with mitoxantrone.^13^ Patients enrolled onto CALGB 10201 received induction chemotherapy consisting of cytarabine and daunorubicin, with or without the *BCL2* antisense oblimersen sodium. The consolidation therapy included two cycles of HiDAC with or without oblimersen sodium.^14^ In CALGB 10502, bortezomib was added to induction consisting of daunorubicin and cytarabine and consolidation with high-dose cytarabine.^15^ Patients enrolled onto CALGB 11002 received decitabine with or without addition of the proteasome inhibitor bortezomib, for both induction and postremission therapy.^16^ For patients treated on CALGB 11001, sorafenib was added to the induction and consolidation treatment consisting of daunorubicin and cytarabine and consolidation with high-dose cytarabine, followed by sorafenib maintenance.^17^

*Patients treated on* *German AML Cooperative Group (AMLCG) protocols*

A total of 954 adult patients with AML treated on AMLCG frontline protocols AMLCG-1999 and AMLCG-2008 were included as validation cohort into the analyses.

In the AMLCG-1999 trial^18^ (clinicaltrials.gov identifier NCT00266136; recruitment period, 1999 to 2011; patients included in this analysis were recruited between 1999 and 2005), patients <60 years were randomized to receive double induction with either one cycle of TAD-9 followed by one cycle of HAM on day 21 (TAD-HAM), or two cycles of HAM 21 days apart (HAM-HAM; TAD-9: thioguanine 100 mg/m2 twice daily on days 3-9, cytarabine 100 mg/m²/d continuous infusion on days 1+2 and 100 mg/m² twice daily on days 3-8, and daunorubicin 60 mg/m² on days 3-5; HAM: cytarabine 3 g/m² twice daily on days 1-3 and mitoxantrone 10 mg/m² on days 3-5). From 2002, 10% of patients aged ≤60 years (n=14 patients in this analysis) were randomized to a common standard arm of the German AML intergroup and received 7+3 induction therapy (cytarabine 100 mg/m²/d continuous infusion on days 1-7 and daunorubicin 60 mg/m² on days 3-5). For postremission treatment patients <60 years underwent upfront randomization to undergo consolidation either with one cycle of TAD-9 followed by three years of monthly cytarabine-based maintenance chemotherapy, or a single TAD-9 consolidation course followed by autologous HSCT and no maintenance. Per the study protocol, all patients aged <60 years with HLA-matched sibling donors were to be offered allogeneic HSCT in first CR, irrespective of cytogenetic or molecular risk factors. Patients ≥60 years were randomized to receive induction therapy with one cycle of either TAD-9 or HAM, followed by a second HAM cycle on day 21 only if ≥5% residual blasts were present in the BM on day 16. All patients ≥60 years were to receive one cycle of TAD-9 consolidation followed by maintenance therapy.

For patients treated on the AMLCG-2008 trial^19^ (NCT01382147; recruitment period, 2009 to 2012), patients <60 years and fit patients up to the age of 70 (‘younger’ patients) were randomized to receive either double induction chemotherapy with TAD-9 and HAM (21 days apart), or dose-dense induction therapy according to the sHAM regimen (cytarabine 3 g/m² [1 g/m² in patients ≥60 years] twice daily on days 1,2,8 and 9; and mitoxantrone 10 mg/m² on days 3,4,10 and 11). Allogeneic HSCT from an HLA-matched related or unrelated donor was the recommended postremission therapy for all younger patients achieving CR except those with favorable genetic features (defined as favorable cytogenetics or cytogenetically normal patients with mutated *NPM1* and no *FLT3*-ITD) and good response to induction chemotherapy (<10% blasts in a bone marrow aspirate obtained on d16 after start of induction therapy). For younger patients without a donor, those unable or unwilling to undergo allogeneic HSCT, and those with a favorable risk profile, postremission therapy consisted of one cycle of TAD-9 for consolidation, followed by 3 years of cytarabine-based maintenance therapy.

Less fit patients aged ≥60 years, and all patients aged ≥70 years, were randomized to receive induction therapy according to the HAM regimen (cytarabine, 1g/m² per dose) followed by a second HAM induction cycle on day 21 only if a bone marrow aspirate on day 16 showed ≥5% blasts, or to dose-dense induction with sHAM (cytarabine, 1g/m² per dose). Postremission therapy in this group consisted of one cycle of TAD-9 for consolidation, followed by 3 years of maintenance therapy.

**Cytogenetic analyses**

For all CALGB/Alliance patients, pretreatment cytogenetic analyses of bone marrow (BM) and/or blood samples were performed by institutional cytogenetics laboratories using unstimulated short-term (24- and/or 48-hour) cultures, and the results were confirmed by central karyotype review as previously reported.^20^ In each patient with cytogenetically normal AML, at least 20 BM metaphase cells were analyzed and the karyotype found to be normal.

**Mutational profiling**

The mutational status of 80 protein coding genes was determined by targeted amplicon sequencing using the MiSeq platform (Illumina, San Diego, CA).^21^ DNA library preparations were performed according to the manufacturer’s instructions. Briefly, samples were pooled and run on the MiSeq machine using the Illumina MiSeq Reagent Kit v3. Sequenced reads were aligned to the hg19 genome build using the Illumina Isis Banded Smith-Waterman aligner. Single nucleotide variant and indel calling were performed using MuTect and VarScan, respectively.^22,23^ The MuCor algorithm was used as the baseline for integrative mutation assessment.^24^ We only considered non-synonymous variants not listed in either the 1000 Genome database or dbSNP142-common variants. All called variants underwent visual inspection of the aligned reads using the Integrative Genomics Viewer (Broad Institute).^25^ All variants that occurred with VAFs of <.02 or were sequenced to a depth of <15 reads were excluded from the analysis. In addition, variants were excluded when they occurred only in one read direction if sequenced in both directions, if the region contained many variants with low quality scores, or if they occurred in all analyzed samples including run controls. In addition, samples with high background noise were entirely excluded from analysis. Samples were considered non-evaluable for a specific gene if ≥85% of the amplicons covering the target regions within the coding sequence of the gene were sequenced to a depth of <15 reads. If <15 reads were present, the gene mutation status was considered as not evaluable.

**Gene expression and differential splicing analyses**

RNA-Seq FASTQ files from the Alliance cohort were aligned to the human genome (hg38) using the software HISAT2.^26^ Gene expression RPKM values and alternative splicing Percent Spliced In (PSI) estimates were determined from the aligned BAM files in AltAnalyze (version 2.1.4, MultiPath-PSI algorithm)^27^ using Ensembl version 91 as a reference. These were compared to previously computed alternative splicing events with Leucegene and BEAT-AML using the same computational pipeline and reference genome/transcriptome.^28^ For all subtype comparisons, samples within each assigned AML subtype were compared to all other AML patients. In addition to the above-described genotypes and clinical subtypes, we performed initial QC on all initial subtype groups by computing differential splicing in AltAnalyze (MultiPath-PSI, dPSI>0.1 and eBayes t-test *P*<0.05, FDR corrected) with expression clustering of the top 200 splicing events (automatePSIClustering function in AltAnalyze) to identify outlier samples that do or do not co-cluster (e.g., low variant allele frequency (VAF), unknown variant impacts). This resulted in a small number of re-assignments for *SF3B1* and *SRSF2* mutations, further informed by mutation burden and VAF. Final differential gene and splicing analyses were performed on these cohort groups. Subtype associated differentially expressed genes were defined as those with a fold>1.5 and eBayes t-test *P*<0.05, FDR corrected. For all sex-based comparisons, male samples were compared to females using the same above thresholds, without FDR correction, due to the limited sample group sizes. Only AML-variant or clinically defined AML subtypes with at least 18 patients were considered for these sex-based comparisons, which removed *U2AF1-Q157*, SF1, SF3A1,* *KMT2A* (*MLL*) partial tandem duplication *(KMT2A-*PTD*)* and *ZRSR2* variant-defined subtypes from comparison. For gene set enrichment, we used the GO-Elite software in AltAnalyze^29^ using default options, and a previously described custom human BM cell-type marker database (top-100 genes per signature).^30^ Visualized networks were produced from GO-Elite. Differentially expressed genes, splicing events and GO-Elite gene set enrichment results have been deposited at Synapse.org

(https://www.synapse.org/#!Synapse:syn52278016).

**Definition of clinical endpoints**

For Alliance data, clinical endpoints were defined according to generally accepted criteria^31-34^ and according to treatment study protocols. Early death was defined as death within 30 days of starting therapy, regardless of cause. Relapse was defined by ≥5% BM blasts, circulating leukemic blasts, or the development of extramedullary leukemia.^34^ Disease-free survival was measured from the date of CR until the date of relapse or death; patients alive and relapse-free at last follow-up were censored. Overall survival was measured from the date on study until the date of death, and patients alive at last follow-up were censored.

**Outcome analyses**

In our outcome analyses, we used *P*-values adjusted to control for per family error rate (probability of a Type I error) for all variables considered in univariable analyses. The families were all variables considered in each outcome analysis and only the variables whose likelihood ratio test adjusted *P*-value was <.20 from the univariable models were considered in the multivariable analysis.

**Supplementary references**

1. Kolitz JE, George SL, Marcucci G, Vij R, Powell BL, Allen SL, et al. P-glycoprotein inhibition using valspodar (PSC-833) does not improve outcomes for patients under age 60 years with newly diagnosed acute myeloid leukemia: Cancer and Leukemia Group B study 19808. Blood. 2010;116:1413-21.
2. Blum W, Sanford BL, Klisovic R, DeAngelo DJ, Uy G, Powell BL, et al. Maintenance therapy with decitabine in younger adults with acute myeloid leukemia in first remission: a phase 2 Cancer and Leukemia Group B study (CALGB 10503). Leukemia. 2017;31:34-9.
3. Kolitz JE, George SL, Dodge RK, Hurd DD, Powell BL, Allen SL, et al. Dose escalation studies of cytarabine, daunorubicin, and etoposide with and without multidrug resistance modulation with PSC-833 in untreated adults with acute myeloid leukemia younger than 60 years: final induction results of Cancer and Leukemia Group B study 9621. J Clin Oncol. 2004;22:4290-301.
4. Mayer RJ, Davis RB, Schiffer CA, Berg DT, Powell BL, Schulman P, et al. Intensive postremission chemotherapy in adults with acute myeloid leukemia. N Engl J Med. 1994;331:896-903.
5. Moore JO, George SL, Dodge RK, Amrein PC, Powell BL, Kolitz JE, et al. Sequential multiagent chemotherapy is not superior to high-dose cytarabine alone as postremission intensification therapy for acute myeloid leukemia in adults under 60 years of age: Cancer and Leukemia Group B study 9222. Blood. 2005;105:3420-7.
6. Stone RM, Mandrekar SJ, Sanford BL, Laumann K, Geyer S, Bloomfield CD, et al. Midostaurin plus chemotherapy for acute myeloid leukemia with a FLT3 mutation. N Engl J Med. 2017;377:454-64.
7. Schiffer CA, Davis RB, Schulman P, et al. Intensive post remission therapy of acute myeloid leukemia (AML) with cytoxan/etoposide (CY/VP16) and diazaquone/mitoxantrone (AZQ/MITO). Blood. 1991;78(suppl):460 (abstract 1829).
8. Moore JO, Dodge RK, Amrein PC, Kolitz J, Lee EJ, Powell B, et al. Granulocyte-colony stimulating factor (filgrastim) accelerates granulocyte recovery after intensive postremission chemotherapy for acute myeloid leukemia with aziridinyl benzoquinone and mitoxantrone: Cancer and Leukemia Group B study 9022. Blood. 1997;89:780-8.
9. Cassileth PA, Harrington DP, Appelbaum FR, Lazarus HM, Rowe JM, Paietta E, et al. Chemotherapy compared with autologous or allogeneic bone marrow transplantation in the management of acute myeloid leukemia in first remission. New Engl J Med. 1998;339:1649-56.
10. Farag SS, George SL, Lee EJ, Baer M, Dodge RK, Becknell B, et al. Postremission therapy with low-dose interleukin 2 with or without intermediate pulse dose interleukin 2 therapy is well tolerated in elderly patients with acute myeloid leukemia: Cancer and Leukemia Group B study 9420. Clin Cancer Res. 2002;8:2812-9.
11. Baer MR, George SL, Caligiuri MA, Sanford BL, Bothun SM, Mrózek K, et al. Low-dose interleukin-2 immunotherapy does not improve outcome of patients age 60 years and older with acute myeloid leukemia in first complete remission: Cancer and Leukemia Group B study 9720. J Clin Oncol. 2008;26:4934-9.
12. Baer MR, George SL, Sanford BL, Mrózek K, Kolitz JE, Moore JO, et al. Escalation of daunorubicin and addition of etoposide in the ADE regimen in acute myeloid leukemia patients aged 60 years and older: Cancer and Leukemia Group B study 9720. Leukemia. 2011;25:800-7.
13. Stone RM, Berg DT, George SL, Dodge RK, Paciucci PA, Schulman P, et al. Granulocyte-macrophage colony stimulating factor after initial chemotherapy for elderly patients with primary acute myelogenous leukemia. N Engl J Med. 1995;332:1671-7.
14. Walker AR, Marcucci G, Yin J, Blum W, Stock W, Kohlschmidt J, et al. Phase 3 randomized trial of chemotherapy with or without oblimersen in older AML patients: CALGB 10201 (Alliance). Blood Adv. 2021;5:2775-87.
15. Attar EC, Johnson JL, Amrein PC, Lozanski G, Wadleigh M, DeAngelo DJ, et al. Bortezomib added to daunorubicin and cytarabine during induction therapy and to intermediate-dose cytarabine for consolidation in patients with previously untreated acute myeloid leukemia age 60 to 75 years: CALGB (Alliance) study 10502. J Clin Oncol. 2012;31:923-9.
16. Roboz GJ, Mandrekar SJ, Desai P, Laumann K, Walker AR, Wang ES, et al. A randomized trial of 10 days of decitabine alone or with bortezomib in previously untreated older patients with acute myeloid leukemia: CALGB 11002 (Alliance). Blood Adv. 2018;2:3608-17.
17. Uy GL, Mandrekar SJ, Laumann K, Marcucci G, Zhao W, Levis MJ, et al. A phase 2 study incorporating sorafenib into the chemotherapy for older adults with FLT3-mutated acute myeloid leukemia: CALGB 11001. Blood Adv. 2017;1:331-40.
18. Stelljes M, Beelen DW, Braess J, Sauerland MC, Heinecke A, Berning B, et al. Allogeneic transplantation as post-remission therapy for cytogenetically high-risk acute myeloid leukemia: landmark analysis from a single prospective multicenter trial. Haematologica. 2011;96:972-9.
19. Braess J, Spiekermann K, Staib P, Grüneisen A, Wörmann B, Ludwig WD, et al. Dose-dense induction with sequential high-dose cytarabine and mitoxantone (S-HAM) and pegfilgrastim results in a high efficacy and a short duration of critical neutropenia in de novo acute myeloid leukemia: a pilot study of the AMLCG. Blood. 2009;113:3903-10.
20. Mrózek K, Carroll AJ, Maharry K, Rao KW, Patil SR, Pettenati MJ, et al. Central review of cytogenetics is necessary for cooperative group correlative and clinical studies of adult acute leukemia: the Cancer and Leukemia Group B experience. Int J Oncol. 2008;33:239-44.
21. Eisfeld AK, Mrózek K, Kohlschmidt J, Nicolet D, Orwick S, Walker CJ, et al. The mutational oncoprint of recurrent cytogenetic abnormalities in adult patients with *de novo* acute myeloid leukemia. Leukemia. 2017;31:2211-8.
22. Cibulskis K, Lawrence MS, Carter SL, Sivachenko A, Jaffe D, Sougnez C, et al. Sensitive detection of somatic point mutations in impure and heterogeneous cancer samples. Nat Biotechnol. 2013;31:213-9.
23. DePristo MA, Banks E, Poplin R, Garimella KV, Maguire JR, Hartl C, et al. A framework for variation discovery and genotyping using next-generation DNA sequencing data. Nat Genet. 2011;43:491-8.
24. Kroll KW, Eisfeld A-K, Lozanski G, Bloomfield CD, Byrd JC, Blachly JS. MuCor: mutation aggregation and correlation. Bioinformatics. 2016;32:1557-8.
25. Robinson JT, Thorvaldsdóttir H, Winckler W, Guttman M, Lander ES, Getz G, et al. Integrative genomics viewer. Nat Biotechnol. 2011;29:24-6.
26. Kim D, Paggi JM, Park C, Bennett C, Salzberg SL. Graph-based genome alignment and genotyping with HISAT2 and HISAT-genotype. Nat Biotechnol. 2019;37:907-15.
27. Fenix AM, Miyaoka Y, Bertero A, Blue SM, Spindler MJ, Tan KKB, et al. Gain-of-function cardiomyopathic mutations in RBM20 rewire splicing regulation and re-distribute ribonucleoprotein granules within processing bodies. Nat Commun. 2021;12:6324.
28. Itskovich SS, Gurunathan A, Clark J, Burwinkel M, Wunderlich M, Berger MR, et al. MBNL1 regulates essential alternative RNA splicing patterns in MLL-rearranged leukemia. Nat Commun. 2020;11:2369.
29. Zambon AC, Gaj S, Ho I, Hanspers K, Vranizan K, Evelo CT, et al. GO-Elite: a flexible solution for pathway and ontology over-representation. Bioinformatics. 2012;28:2209-10.
30. Hay SB, Ferchen K, Chetal K, Grimes HL, Salomonis N. The Human Cell Atlas bone marrow single-cell interactive web portal. Exp Hematol. 2018;68:51-61.
31. Döhner H, Estey EH, Amadori S, Appelbaum FR, Büchner T, Burnett AK, et al. Diagnosis and management of acute myeloid leukemia in adults: recommendations from an international expert panel, on behalf of the European LeukemiaNet. Blood. 2010;115:453-74.
32. Döhner H, Estey E, Grimwade D, Amadori S, Appelbaum FR, Büchner T, et al. Diagnosis and management of AML in adults: 2017 ELN recommendations from an international expert panel. Blood. 2017;129:424-47.
33. Döhner H, Wei AH, Appelbaum FR, Craddock C, DiNardo CD, Dombret H, et al. Diagnosis and management of AML in adults: 2022 recommendations from an international expert panel on behalf of the ELN. Blood. 2022;140:1345-77.
34. Cheson BD, Cassileth PA, Head DR, Schiffer CA, Bennett JM, Bloomfield CD, et al. Report of the National Cancer Institute-sponsored workshop on definitions of diagnosis and response in acute myeloid leukemia. J Clin Oncol. 1990;8:813-9.

**Supplementary Table 1.** Comparison of pretreatment clinical and cytogenetic characteristics of female and male patients with AML, treated on CALGB/Alliance or AMLCG protocols.

| **Characteristic** | **CALGB/Alliance Patients** | | | **AMLCG Patients** | | |
| --- | --- | --- | --- | --- | --- | --- |
|  | **Female**  **n=749** | **Male**  **n=977** | ***P**** | **Female**  **n=465** | **Male**  **n=489** | ***P**** |
| Age, y |  |  | 0.06 |  |  | 0.19 |
| Median   Range | 51  17-92 | 54  17-86 |  | 55  18-86 | 58  18-85 |  |
| Race, *n* (%) |  |  | 0.30 | Not available |  |  |
| White | 636 (86) | 845 (88) |  |  |  |  |
| Non-white | 100 (14) | 113 (12) |  |  |  |  |
| Hemoglobin, g/dL |  |  | 0.21 |  |  | 0.98 |
| Median   Range | 9.2  2.9-25.1 | 9.2  2.3-15.8 |  | 9.1  3.6-13.9 | 9.0  3.5-16.0 |  |
| Platelet count, x10^9^/L |  |  | 0.99 |  |  | 0.08 |
| Median   Range | 55  5-989 | 55  4-850 |  | 59  0-1760 | 52  0-670 |  |
| White blood cell count, x10^9^/L |  |  | 0.009 |  |  | 0.07 |
| Median   Range | 26.0  0.4-560.0 | 22.2  0.4-475.0 |  | 25.0  0.1-798.2 | 18.9  0.4-666.0 |  |
| Bone marrow blasts, % |  |  | 0.04 |  |  | 0.23 |
| Median   Range | 68  0-98 | 65  0-99 |  | 80  10-100 | 80  9-100 |  |
| 2022 ELN genetic group, *n* (%) |  |  | <0.001 | Not available |  |  |
| Favorable | 271 (38) | 314 (36) |  |  |  |  |
| Intermediate | 213 (30) | 180 (20) |  |  |  |  |
| Adverse | 222 (31) | 388 (44) |  |  |  |  |
| CN-AML, *n* (%) |  |  | <0.001 |  |  | 0.002 |
| Yes | 389 (52) | 406 (42) |  | 329 (71) | 294 (62) |  |
| No | 360 (48) | 571 (58) |  | 132 (29) | 183 (38) |  |
| Complex karyotype, *n* (%) |  |  | 0.005 |  |  | 0.42 |
| Yes | 58 (8) | 116 (12) |  | 36 (8) | 45 (9) |  |
| No | 691 (92) | 861 (88) |  | 426 (92) | 433 (91) |  |
| t(8;21)(q22;q22), *n* (%) |  |  | 0.80 |  |  | 0.11 |
| Yes | 28 (4) | 40 (4) |  | 11 (2) | 21 (4) |  |
| No | 721 (96) | 937 (96) |  | 451 (98) | 456 (96) |  |
| inv(16)(p13.1q22), *n* (%) |  |  | 0.55 |  |  | 0.27 |
| Yes | 44 (6) | 65 (7) |  | 17 (4) | 25 (5) |  |
| No | 705 (94) | 912 (93) |  | 445 (96) | 452 (95) |  |
| 11q23*/KMT2A*-rearranged AML, *n* (%) |  |  | 0.21 |  |  | 1.00 |
| Yes | 49 (7) | 49 (5) |  | 16 (3) | 16 (3) |  |
| No | 700 (93) | 928 (95) |  | 446 (97) | 461 (97) |  |

CN-AML, cytogenetically normal AML; ELN, European LeukemiaNet; NA. not available.

***** *P*-values are from the Wilcoxon rank sum test for continuous variables and from the Fisher’s exact test for categorical variables and compare the two groups: female and male patients with AML.

**Supplementary Table 2.** Outcomes of female and male patients with AML aged <60 years treated on frontline CALGB/Alliance study protocols and of those treated on AMLCG protocols.

| **Endpoint** | **Female Patients** | **Male Patients** | ***P**** |
| --- | --- | --- | --- |
| **CALGB/Alliance patients** | **n=381** | **n=463** |  |
| Early death, n (%) | 26 (7) | 33 (7) | 0.89 |
| Complete remission, n (%) | 298 (78) | 338 (73) | 0.09 |
| Disease-free survival  Median, y  Disease-free at 5 y, % (95% CI) | 1.6   40 (34-45) | 1.7   39 (34-44) | 0.91 |
| Overall survival  Median, y  Alive at 5 y, % (95% CI) | 1.9   38 (33-43) | 1.5   35 (31-39) | 0.48 |
| **AMLCG patients** | **n=283** | **n=257** |  |
| Early death, n (%) | 11 (4) | 17 (7) | 0.18 |
| Complete remission, n (%) | 215 (76) | 184 (72) | 0.28 |
| Disease-free survival  Median, y  Disease-free at 5 y, % (95% CI) | 5.0   51 (43-57) | 2.2   46 (38-54) | 0.17 |
| Overall survival  Median, y  Alive at 5 y, % (95% CI) | 8.8   51 (45-57) | 1.8   42 (36-48) | 0.005 |

* *P*-values for early death and complete remission are from Fisher’s exact test, *P*-values for the time to event variables are from the log-rank test and compare the two groups: female and male patients with AML.

**Supplementary Table 3.** Final multivariable outcome analyses of female and male patients with AML younger than <60 years treated on the CALGB/Alliance study protocols.

| **Female patients** | | | | **Male patients** | | | |  | | | | |  | | | | |  | | | |  | |  | | | |  | |  | |  | |  |  |
| --- | --- | --- | --- | --- | --- | --- | --- | --- | --- | --- | --- | --- | --- | --- | --- | --- | --- | --- | --- | --- | --- | --- | --- | --- | --- | --- | --- | --- | --- | --- | --- | --- | --- | --- | --- |
| **Variable** | **Categories** | ***P**** | **Hazard ratio/**  **odds ratio**  **(95% CI)** | **Variable** | **Categories** | ***P**** | **Hazard ratio/**  **odds ratio**  **(95% CI)** |  | | | | |  | | | | |  | | | |  | |  | | | |  | |  | |  | |  |  |
| **Complete remission attainment** | | | | | | | |  | | | | |  | | | | |  | | | |  | |  | | | |  | |  | |  | |  |  |
| *NPM1* | Mutated vs wild-type | <0.001 | 3.05 (1.64-5.68) | *NPM1* | Mutated vs wild-type | 0.02 | 2.60 (1.18-5.71) |  | | | | |  | | | | |  | | | |  | |  | | | |  | |  | |  | |  |  |
| *CEBPA* | bZIP vs wild-type or other | 0.02 | 11.53 (1.50-88.73) | *CEBPA* | bZIP vs wild-type or other | 0.02 | 6.24 (1.33-29.20) |  | | | | |  | | | | |  | | | |  | |  | | | |  | |  | |  | |  |  |
| CBF-AML | Yes vs no | 0.002 | 7.45 (2.11-26.28) | CBF-AML | Yes vs no | <0.001 | 13.57 (4.52-40.81) |  | | | | |  | | | | |  | | | |  | |  | | | |  | |  | |  | |  |  |
| *FLT3*-ITD | Yes vs no | 0.02 | 0.46 (0.25-0.87) | *SF3B1* | Mutated vs wild-type | 0.001 | 0.14 (0.04-0.48) |  | | | | |  | | | | |  | | | |  | |  | | | |  | |  | |  | |  |  |
| *PTPN11* | Mutated vs wild-type | 0.02 | 0.37 (0.17-0.83) | CN-AML | Yes vs no | 0.001 | 3.44 (1.63-7.26) |  | | | | |  | | | | |  | | | |  | |  | | | |  | |  | |  | |  |  |
| Complex karyotype | Yes vs no | 0.006 | 0.20 (0.06-0.63) | Hemoglobin | Continuous | 0.007 | 1.24 (1.06-1.45) |  | | | | |  | | | | |  | | | |  | |  | | | |  | |  | |  | |  |  |
| Platelets | Continuous, 50-unit increase | 0.02 | 1.38 (1.05-1.81) | WBC | Continuous, 50-unit increase | 0.04 | 0.78 (0.62-0.99) |  | | | | |  | | | | |  | | | |  | |  | | | |  | |  | |  | |  |  |
| **Disease-free survival** | | | | | | | |  | | | | |  | | | | |  | | | |  | |  | | | |  | |  | |  | |  |  |
| *FLT3*-ITD | Yes vs no | 0.003 | 1.70 (1.20-2.39) | *FLT3*-ITD | Yes vs no | 0.003 | 1.71 (1.20-2.44) |  | | | | |  | | | | |  | | | |  | |  | | | |  | |  | |  | |  |  |
| CBF-AML | Yes vs no | 0.006 | 0.48 (0.28-0.81) | CBF-AML | Yes vs no | <0.001 | 0.36 (0.22-0.60) |  | | | | |  | | | | |  | | | |  | |  | | | |  | |  | |  | |  |  |
| *FLT3*-TKD | Yes vs no | 0.02 | 0.59 (0.39-0.91) | *CEBPA* | bZIP vs wild-type or other | 0.007 | 0.50 (0.31-0.83) |  | | | | |  | | | | |  | | | |  | |  | | | |  | |  | |  | |  |  |
| *RUNX1* | Mutated vs wild-type | 0.03 | 1.89 (1.05-3.38) | Complex karyotype | Yes vs no | <0.001 | 3.43 (2.00-5.89) |  | | | | |  | | | | |  | | | |  | |  | | | |  | |  | |  | |  |  |
| *WT1* | Mutated vs wild-type | <0.001 | 2.46 (1.61-3.76) | Platelets | Continuous, 50-unit increase | 0.02 | 0.85 (0.74-0.98) |  | | | | |  | | | | |  | | | |  | |  | | | |  | |  | |  | |  |  |
| *DNMT3A* | Mutated vs wild-type | 0.03 | 1.46 (1.04-2.05) | Age | Continuous, 10-year increase | 0.04 | 1.15 (1.01-1.32) |  | | | | |  | | | | |  | | | |  | |  | | | |  | |  | |  | |  |  |
| Hemoglobin | Continuous | 0.006 | 0.89 (0.82-0.97) | Extramedullary Involvement | Yes vs no | 0.01 | 0.63 (0.74-0.90) |  | | | | |  | | | | |  | | | |  | |  | | | |  | |  | |  | |  |  |
| WBC | Continuous, 50-unit increase | 0.002 | 1.26 (1.09-1.46) |  |  |  |  |  | | | | |  | | | | |  | | | |  | |  | | | |  | |  | |  | |  |  |
|  |  |  |  |  |  |  |  |  | | | | |  | | | | |  | | | |  | |  | | | |  | |  | |  | |  |  |
| **Overall survival** | | | | | | | |  |  |  | |  | |  | |  |  | |  |  | | |  | | |  | | |  | | Yes vs no | | <0.0041 | | 1.55 (1.15, 2.08)2.04 (1.50-2.78) |
| *TP53* | Mutated vs wild-type | 0.004 | 2.38 (1.32-4.32) | *TP53* | Mutated vs wild-type | 0.003 | 1.99 (1.26-3.16) |  | | |  | | | |  | | |  | | |  | |  | |  | |  | |  | |  | |  | |  |
| *FLT3*-ITD | Yes vs no | <0.001 | 1.74 (1.31-2.31) | *FLT3*-ITD | Yes vs no | 0.004 | 1.55 (1.15-2.08) |  | | |  | | | |  | | |  | | |  | |  | |  | |  | |  | |  | |  | | *FLT3*-ITD |
| CBF-AML | Yes vs no | 0.03 | 0.59 (0.36-0.96) | CBF-AML | Yes vs no | <0.001 | 0.22 (0.14-0.37) |  | | | | |  | | | | |  | | | |  | |  | | | |  | |  | |  | |  |  |
| Complex karyotype | Yes vs no | <0.001 | 3.60 (2.00-6.50) | Complex karyotype | Yes vs no | <0.001 | 2.07(1.37-3.14) |  | | | | |  | | | | |  | | | |  | |  | | | |  | |  | |  | |  |  |
| WBC | Continuous, 50-unit increase | <0.001 | 1.25 (1.13-1.39) | WBC | Continuous, 50-unit increase | 0.01 | 1.15 (1.03-1,27) |  | | | | |  | | | | |  | | | |  | |  | | | |  | |  | |  | |  |  |
| Age | Continuous, 10-year increase | <0.001 | 1.26 (1.11-1.43) | Age | Continuous, 10-year increase | 0.04 | 1.13 (1.01-1.26) |  | | | | |  | | | | |  | | | |  | |  | | | |  | |  | |  | |  |  |
| *RUNX1* | Mutated vs wild-type | 0.006 | 1.88 (1.20-2.93) | *SF3B1* | Mutated vs wild-type | 0.003 | 2.32 (1.33-4.05) |  | | | | |  | | | | |  | | | |  | |  | | | |  | |  | |  | |  |  |
| *WT1* | Mutated vs wild-type | <0.001 | 1.87 (1.30-2.70) | *NPM1* | Mutated vs wild-type | <0.001 | 0.53 (0.40-0.71) |  | | | | |  | | | | |  | | | |  | |  | | | |  | |  | |  | |  |  |
|  |  |  |  | *CEBPA* | bZIP vs wild-type or other | <0.001 | 0.34 (0.21-0.54) |  | | | | |  | | | | |  | | | |  | |  | | | |  | |  | |  | |  |  |

CBF-AML, core-binding factor AML; CN-AML, cytogenetically normal AML; *FLT3*-ITD, internal tandem duplication of the *FLT3* gene; *FLT3*-TKD, mutation in the tyrosine kinase domain of the *FLT3* gene; HR, hazard ratio; OR, odds ratio.

The following variables, with *P*≤.20 in univariable analyses, were considered in multivariable models:

**Female patients: complete remission attainment:** mutations in *BCOR*, *CEBPA*, *FLT3*-ITD, *FLT3*-TKD, *IDH2*, *NPM1*, *NRAS*, *PTPN11*, *RUNX1*, *SAMHD1*, *SMC1A*, *TP53*, presence of complex karyotype, CBF-AML, continuous count of platelets, white blood cell; **disease-free survival.** mutations in *AKT1, CCND2, DNMT3A, FLT3*-ITD*, FLT3*-TKD*, IDH1, RUNX1, TYK2, WT1,* presence of complex karyotype, CBF-AML, continuous count of Hemoglobin, white blood cell, age; **overall survival.** mutations in *BCOR, CCND2, CEBPA, FLT3*-ITD*, FLT3*-TKD*, DNMT3A, EZH2, IDH1, IDH2, KIT, RUNX1, SMC1A, TET2, TP53, TYK2, U2AF1, WT1,* presence of complex karyotype, CBF-AML, continuous count of hemoglobin, platelets, white blood cell, age.

**Male patients: complete remission attainment:** mutations in ARAF, BRD4, *CEBPA*, *NPM1, RUNX1, SF3B1, SMC3, SRSF2, STAG2, TP53,* presence of cytogenetically normal AML, complex karyotype, CBF-AML, continuous count of hemoglobin, white blood cell, age; **disease-free survival.** mutations in *ASXL1, BCOR, CEBPA, DNMT3A, FLT3*-ITD*, GAT2, IDH2, NRAS, RAD21, RUNX1, SMARCA2, SRSF2, STAG2, TP53, U2AF1,,* presence of complex karyotype, CBF-AML, continuous count of platelets, age; **overall survival.** mutations in ARAF, *ASXL1, BCOR*, CEBPA, *FLT3*-ITD*, GATA2, KIT, NPM1, NRAS, RUNX1, SF3B1, SRSF2, STAG2, TP53, U2AF1,* presence of cytogenetically normal AML, complex karyotype, CBF-AML, extramedullary involvement, continuous count of white blood cell, age

.

**Supplementary Table 4.** Outcomes of female and male patients with AML aged ≥60 years treated on frontline CALGB/Alliance study protocols and of those treated on AMLCG protocols.

| **Endpoint** | **Female Patients** | **Male Patients** | ***P**** |
| --- | --- | --- | --- |
| **CALGB/Alliance patients** | **n=220** | **n=304** |  |
| Early death, n (%) | 45 (20) | 53 (17) | 0.43 |
| Complete remission, n (%) | 96 (44) | 145 (48) | 0.38 |
| Disease-free survival  Median, y  Disease-free at 3 y, % (95% CI) | 0.6   17 (10-25) | 0.6   9 (5-14) | 0.08 |
| Overall Survival  Median, y  Alive at 3 y, % (95% CI) | 0.5  12 (8-16) | 0.6   8 (5-12) | 0.60 |
| **AMLCG patients** | **n=182** | **n=232** |  |
| Early death, n (%) | 13 (7) | 29 (13) | 0.10 |
| Complete remission, n (%) | 121 (66) | 148 (64) | 0.60 |
| Disease-free survival  Median, y  Disease-free at 3 y, % (95% CI) | 0.6   25 (17-33) | 1.1   29 (21-37) | 0.23 |
| Overall Survival  Median, y  Alive at 3 y, % (95% CI) | 1.0   28 (21-34) | 1.1   31 (25-37) | 0.75 |

* *P*-values for early death and complete remission are from Fisher’s exact test, *P*-values for the time to event variables are from the log-rank test and compare the two groups: female and male patients with AML.

**Supplementary Table 5.** Final multivariable outcome analyses of female and male patients with AML aged 60 years or older treated on the CALGB/Alliance study protocols.

| **Female patients** | | | | **Male patients** | | | |
| --- | --- | --- | --- | --- | --- | --- | --- |
| **Variable** | **Categories** | ***P**** | **Hazard ratio/**  **odds ratio**  **(95% CI)** | **Variable** | **Categories** | ***P**** | **Hazard ratio/**  **odds ratio**  **(95% CI)** |
| **Complete remission attainment** | | | | | | | |
| *NPM1* | Mutated vs wild-type | 0.02 | 2.28 (1.17-4.44) | *NPM1* | Mutated vs wild-type | <0.001 | 3.16 (1.86-5.39) |
| Complex karyotype | Yes vs no | 0.004 | 0.17 (0.05-0.58) | *TP53* | Mutated vs wild-type | 0.003 | 0.31 (0.15-0.66) |
| WBC | Continuous, 50-unit increase | 0.006 | 0.69 (0.53-0.90) |  |  |  |  |
| Age | Continuous, 10-year increase | <0.001 | 0.39 (0.23-0.67) |  |  |  |  |
| Extramedullary Involvement | Present vs absent | 0.03 | 0.42 (0.20-0.91) |  |  |  |  |
| **Disease-free survival** | | | | | | | |
| *FLT3-*ITD | Mutated vs wild-type | 0.002 | 2.34 (1.35-4.05) | *FLT3-*ITD | Mutated vs wild-type | <0.001 | 2.15 (1.37-3.38) |
| *NPM1* | Mutated vs wild-type | 0.02 | 0.54 (0.32-0.92) | *NPM1* | Mutated vs wild-type | <0.001 | 0.34 (0.22-0.52) |
| *TP53* | Mutated vs wild-type | 0.04 | 2.79 (1.03-7.53) | *PTPN11* | Mutated vs wild-type | <0.001 | 3.05 (1.68-5.53) |
| Extramedullary Involvement | Present vs absent | 0.01 | 2.19 (1.19-4.05) | Complex karyotype | Yes vs no | <0.001 | 3.42 (1.81-6.45) |
|  |  |  |  | WBC | Continuous, 50-unit increase | <0.001 | 1.58 (1.25-1.98) |
| **Overall survival** | | | | | | | |
| *FLT3-*ITD | Mutated vs wild-type | 0.01 | 1.56 (1.11-2.20) | *FLT3-*ITD | Mutated vs wild-type | 0.007 | 1.54 (1.12-2.10) |
| *NPM1* | Mutated vs wild-type | 0.04 | 0.71 (0.50-0.99) | *NPM1* | Mutated vs wild-type | <0.001 | 0.45 (0.34-0.61) |
| Complex karyotype | Yes vs no | <0.001 | 3.12 (1.92-5.07) | Complex karyotype | Yes vs no | 0.02 | 1.59 (1.09-2.31) |
| Age | Continuous, 10-year increase | 0.006 | 1.42 (1.11-1.82) | *KRAS* | Mutated vs wild-type | 0.006 | 3.58 (1.45-8.80) |
| Platelets | Continuous, 50-unit increase | 0.02 | 0.89 (0.81-0.98) | *TP53* | Mutated vs wild-type | <0.001 | 2.39 (1.61-3.55) |
| Extramedullary Involvement | Present vs absent | 0.003 | 1.67 (1.19-2.36) | WBC | Continuous, 50-unit increase | 0.002 | 1.17 (1.06-1.30) |

CBF-AML, core-binding factor AML; CN-AML, cytogenetically normal AML; *FLT3*-ITD, internal tandem duplication of the *FLT3* gene; *FLT3*-TKD, mutation in the tyrosine kinase domain of the *FLT3* gene; HR, hazard ratio; OR, odds ratio.

The following variables, with *P*≤0.20 in univariable analyses, were considered in multivariable models:

**Female patients: complete remission attainment:** mutations in *DNMT3A,* *NPM1*, presence of cytogenetically normal AML, complex karyotype, extramedullary involvement, continuous count of white blood cell, age; **disease-free survival.** mutations in *DNMT3A, FLT3*-ITD*, HIST1H1E, IDH1, NPM1, SF3B1, TP53,* presence of cytogenetically normal AML, extramedullary involvement; **overall survival.** mutations in *ASXL1, CEBPA, FLT3*-ITD*, HIST1H1E, IDH1, NPM1, PICK3CG, TP53, TYK2, U2AF1, WT1,* presence of cytogenetically normal AML, complex karyotype, extramedullary involvement, continuous count of platelets, white blood cell, age.

**Male patients: complete remission attainment:** mutations in BCOR, *IDH1*, *NPM1, TP53, WT1,* presence of cytogenetically normal AML, complex karyotype, continuous count of hemoglobin, age; **disease-free survival.** mutations in *CEBPA, FLT3*-ITD*, IDH2, NPM1, NRAS, PTPN11, SRSF2, TP53, U2AF1,* presence of complex karyotype, continuous count of white blood cell; **overall survival.** mutations in *FLT3*-ITD*, IDH1, IDH2, KIT, KRAS, NPM1, SRSF2, TP53, U2AF1, WT1,* presence of cytogenetically normal AML, complex karyotype, continuous count of hemoglobin, white blood cell, age.

**Supplementary Figure 1.** **Outcomes of female and male patients aged <60 years with AML treated on the CALGB/Alliance** **frontline protocols categorized into the 2022 ELN genetic-risk groups.** **A** Disease-free survival and **B** overall survival of patients.

**A** **B**

**Supplementary Figure 2. Sex-specific survival impact of *WT1* mutations.** **A** Disease-free survival of female and male patients aged <60 years with AML, treated on CALGB/Alliance frontline protocols, who harbored *WT1* mutations and of those who had wild-type *WT1*. **B** Disease-free and **C** overall survival of female and male patients aged <60 years with AML, treated on AMLCG frontline protocols, who harbored *WT1* mutations and of those who had wild-type *WT1*.

**A** **B**

**C**

**Supplementary Figure 3.** **Overall survival of male patients aged <60 years with AML treated on AMLCG frontline protocols who harbored *SF3B1* mutations and of those who did not.** There were not enough female *SF3B1*-mutated patients for analysis.

**Supplementary Figure 4. Outcomes of female and male patients aged ≥60 years with AML treated on the CALGB/Alliance frontline protocols categorized into the 2022 ELN genetic-risk groups.** **A** Disease-free survival and **B** overall survival.

**A** **B**

**Supplementary Figure 5. Global and pathway-level differences in co-existing molecular features of female compared to male patients with acute myeloid leukemia (AML).** **A** Heatmap of the top 150 significant splicing events associated with selected *SF3B1* mutations (top bar) empirically observed to result in similar splicing impacts, for all AML patient samples. **B** Overlap of *SF3B1*-mutant splicing differences (dPSI>0.1 and eBayes t-test *P*<0.05, FDR corrected) in three published AML cohorts. The Alliance cohort had the largest number of *SF3B1*-mutated patients (11 males, 12 females). **C** Heatmap of gene set enrichments (GO-Elite) for differentially expressed genes in male versus female patients in each AML subtype against broad curated biological pathways from the Pathway Commons database. **D** GO-Elite enriched gene network of upregulated genes in male versus female *SF3B1*-mutated patients.


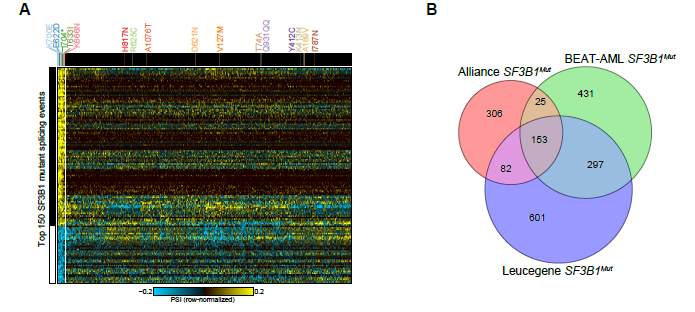


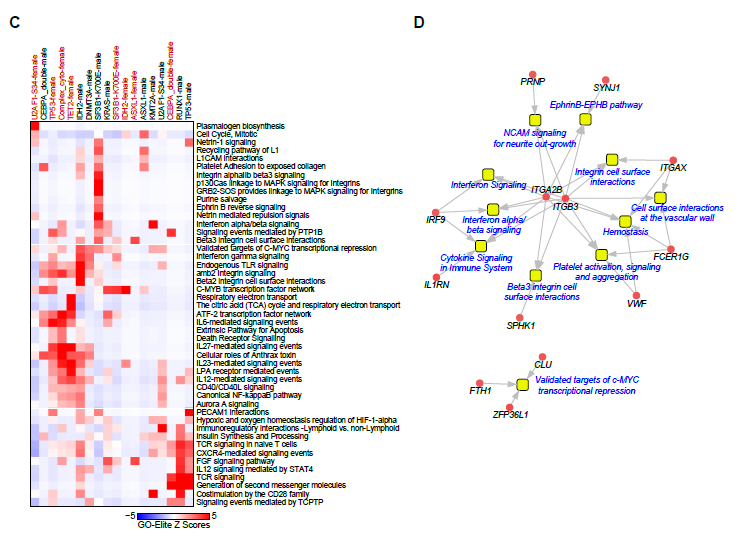

Supplement: Supplementary file 1 — Supplementary Information [file 41375_2023_2068_MOESM1_ESM.docx]
